# Atomistic mechanisms of the regulation of small-conductance Ca^2+^-activated K^+^ channel (SK2) by PIP2

**DOI:** 10.1073/pnas.2318900121

**Published:** 2024-09-17

**Authors:** Ryan L. Woltz, Yang Zheng, Woori Choi, Khoa Ngo, Pauline Trinh, Lu Ren, Phung N. Thai, Brandon J. Harris, Yanxiao Han, Kyle C. Rouen, Diego Lopez Mateos, Zhong Jian, Ye Chen-Izu, Eamonn J. Dickson, Ebenezer N. Yamoah, Vladimir Yarov-Yarovoy, Igor Vorobyov, Xiao-Dong Zhang, Nipavan Chiamvimonvat

**Affiliations:** ^a^Department of Internal Medicine, Division of Cardiovascular Medicine, University of California, Davis, CA 95616; ^b^Department of Physiology and Membrane Biology, University of California, Davis, CA 95616; ^c^Stanford Cardiovascular Institute, Stanford University, Stanford, CA 94305; ^d^Department of Pharmacology, University of California, Davis, CA 95616; ^e^Department of Translational Neuroscience, University of Arizona College of Medicine, Phoenix, AZ 85004; ^f^Department of Anesthesiology and Pain Medicine, University of California, Davis, CA 95616; ^g^Department of Veterans Affairs, Northern California Health Care System, Mather, CA 95655; ^h^Department of Basic Medical Sciences, University of Arizona College of Medicine, Phoenix, AZ 85004

**Keywords:** small conductance Ca^2+^-activated K^+^ channel, phosphatidylinositol 4,5-bisphosphate, optogenetics, calmodulin, atrial arrhythmias

## Abstract

Small-conductance Ca^2+^-activated K^+^ channels (SK, K_Ca_2) are gated solely by intracellular microdomain Ca^2+^. The channel has emerged as a therapeutic target for cardiac arrhythmias. Calmodulin (CaM) interacts with the CaM binding domain (CaMBD) of the SK channels, serving as the obligatory Ca^2+^ sensor to gate the channels. In heterologous expression systems, phosphatidylinositol 4,5-bisphosphate (PIP2) coordinates with CaM in regulating SK channels. However, the roles and mechanisms of PIP2 in regulating SK channels in cardiomyocytes remain unknown. Here, optogenetics, magnetic nanoparticles, combined with *Rosetta* structural modeling, and molecular dynamics (MD) simulations revealed the atomistic mechanisms of how PIP2 works in concert with Ca^2+^-CaM in the SK channel activation. Our computational study affords evidence for the critical role of the amino acid residue R395 in the S6 transmembrane segment, which is localized in propinquity to the intracellular hydrophobic gate. This residue forms a salt bridge with residue E398 in the S6 transmembrane segment from the adjacent subunit. Both R395 and E398 are conserved in all known isoforms of SK channels. Our findings suggest that the binding of PIP2 to R395 residue disrupts the R395:E398 salt bridge, increasing the flexibility of the transmembrane segment S6 and the activation of the channel. Importantly, our findings serve as a platform for testing of structural-based drug designs for therapeutic inhibitors and activators of the SK channel family. The study is timely since inhibitors of SK channels are currently in clinical trials to treat atrial arrhythmias.

## Significance

Atrial fibrillation (AF) is the most common cardiac arrhythmia seen clinically and is associated with a significant increase in morbidity and mortality. Small-conductance Ca^2+^-activated K^+^ channels (SK, K_Ca_2) are gated solely by intracellular Ca^2+^. The channel has emerged as a therapeutic target for AF. However, inhibition of SK channels has been shown to be both antiarrhythmic and proarrhythmic. Therefore, additional insights into the regulatory mechanisms of SK channels are urgently needed. Here, optogenetics, magnetic nanoparticles, structural modeling, and molecular dynamics (MD) simulations revealed the atomistic mechanisms of how phosphatidylinositol 4,5-bisphosphate (PIP2) works in concert with Ca^2+^-calmodulin in the SK channel activation. The study is timely since inhibitors of SK channels are currently in clinical trials to treat cardiac arrhythmias.

Small-conductance Ca^2+^-activated K^+^ (SK, K_Ca_2) channels are unique since they are gated solely by the rise in intracellular Ca^2+^ (Ca^2+^_i_) and provide the direct link between changes in the Ca^2+^_i_ and membrane potentials. The discovery of SK channels dates back more than two decades, with the molecular identification of the channels in the mammalian brain in 1996 (1, 2). The channels are sensitive to the bee venom toxin apamin. The family of SK channels consists of three members with differential sensitivity to apamin: SK1 (or K_Ca_2.1), SK2 (or K_Ca_2.2), and SK3 (or K_Ca_2.3), encoded by *KCNN1*, *2*, and *3* genes, respectively (2, 3). An intermediate-conductance Ca^2+^-activated K^+^ channel (IK, SK4, or K_Ca_2.4 encoded by the *KCNN4* gene) is structurally and functionally similar to the SK channels and is classified to the same gene family (3, 4). Functional SK channels assemble to form homomeric or heteromeric tetramers (1, 5, 6).

We first demonstrated unequivocally that several isoforms of SK channels underlie Ca^2+^-activated K^+^ current (*I*_K,Ca_) in cardiomyocytes(6–8). Subsequently, the field’s knowledge of cardiac SK channels has been dramatically expanded. Our group (2, 6–21) and others (21–35) have substantiated SK channels’ essential roles in the heart. Interests in cardiac SK channels are further fueled by studies suggesting the possible roles of the channels in human arrhythmias and atrial fibrillation (AF) (22–26). The channels may represent a potential therapeutic target for atrial arrhythmias (27–32). Moreover, SK channels are the only known K^+^ channels up-regulated in heart failure (HF) (21, 33–35), underpinning their importance in normal and diseased hearts.

SK channels are highly expressed in atrial compared to ventricular myocytes, and null mutation of the SK2 channels results in atrial arrhythmias and atrioventricular (AV) node dysfunction (10, 11). Different isoforms of SK channels are expressed in human cardiomyocytes, and the channel subunits heteromultimerize via the coiled-coil domains at the C termini (6). Of paramount physiological and clinical importance, we demonstrate that SK currents (*I*_K,Ca_) contribute significantly to the repolarization process in human atria (7, 8). We have uncovered mechanisms underlying SK channel trafficking and functional cross talk between SK and voltage-gated Ca^2+^ channels (Ca_V_) in cardiomyocytes (9, 12, 14, 15). In addition to the direct activation of SK channels by Ca^2+^, an increase in Ca^2+^_i_ seen during AF increases SK channel membrane localization (14).

SK channels are gated by Ca^2+^_i_, localized within their microdomain. The channels sense the rise in Ca^2+^_i_ from Ca^2+^ entry *via* L-type Ca^2+^ channels (LTCCs) and Ca^2+^ release from intracellular stores by the ryanodine receptors (RyR2) (18). Moreover, phosphatidylinositol 4,5-bisphosphate (PIP2) is critical in regulating SK channels (36–38). Polyphosphoinositides are reversibly phosphorylated derivatives of the membrane phospholipid phosphatidylinositol that play critical roles in myriads of cellular function (39). PIP2 is an essential regulator of plasma membrane-bound proteins, including ion channels (40–44). Hence, PIP2 may play critical roles in arrhythmia initiation and maintenance (45, 46). PIP2 may be depleted in HF from sustained phospholipase C (PLC) activation, mediated via G_αq_ protein (45, 46), leading to decreased SK currents. Previous studies in heterologous expression systems demonstrated that PIP2 depletion reduces SK2 currents, and the effects are critically modulated by casein kinase 2 (CK2) phosphorylation of calmodulin (CaM) (36). However, the roles and mechanisms of PIP2 in regulating SK channels in cardiomyocytes remain unknown.

Here, we tested the hypothesis that SK channels are regulated by PIP2 in cardiomyocytes and SK2 channel activation occurs via a two-step process, from the closed to the activated state through the open but nonconductive intermediary, requiring both Ca^2+^ binding to CaM and channel interactions with PIP2. The hypothesis is strongly supported by the recent cryogenic-electron microscopic (cryo-EM) structures of human (h) SK4 (*h*SK4) in closed, open but nonconductive, and open and potentially conductive states (47). Using optogenetics and magnetic nanoparticles and Rosetta structural modeling and molecular dynamics (MD) simulations, we deciphered the roles of PIP2 regulation on SK channels in native cardiomyocytes. We took advantage of the cryo-EM structures of the *h*SK4 channel (47) to develop *h*SK2 atomistic structural models through Rosetta homology modeling and MD simulations to directly test the dynamic regulation of SK channels by Ca^2+^-CaM and PIP2. The study represents critical steps to disentangle the functional roles of SK channels in normal and disease states, focusing on this unique K^+^ channel that compensates for the much needed “repolarization reserve” in HF.

## Results

### Regulation of Apamin-Sensitive Current (*I_K,Ca_*) in Ventricular Myocytes by PIP2 Using Optogenetics.

**Fig. 1.**
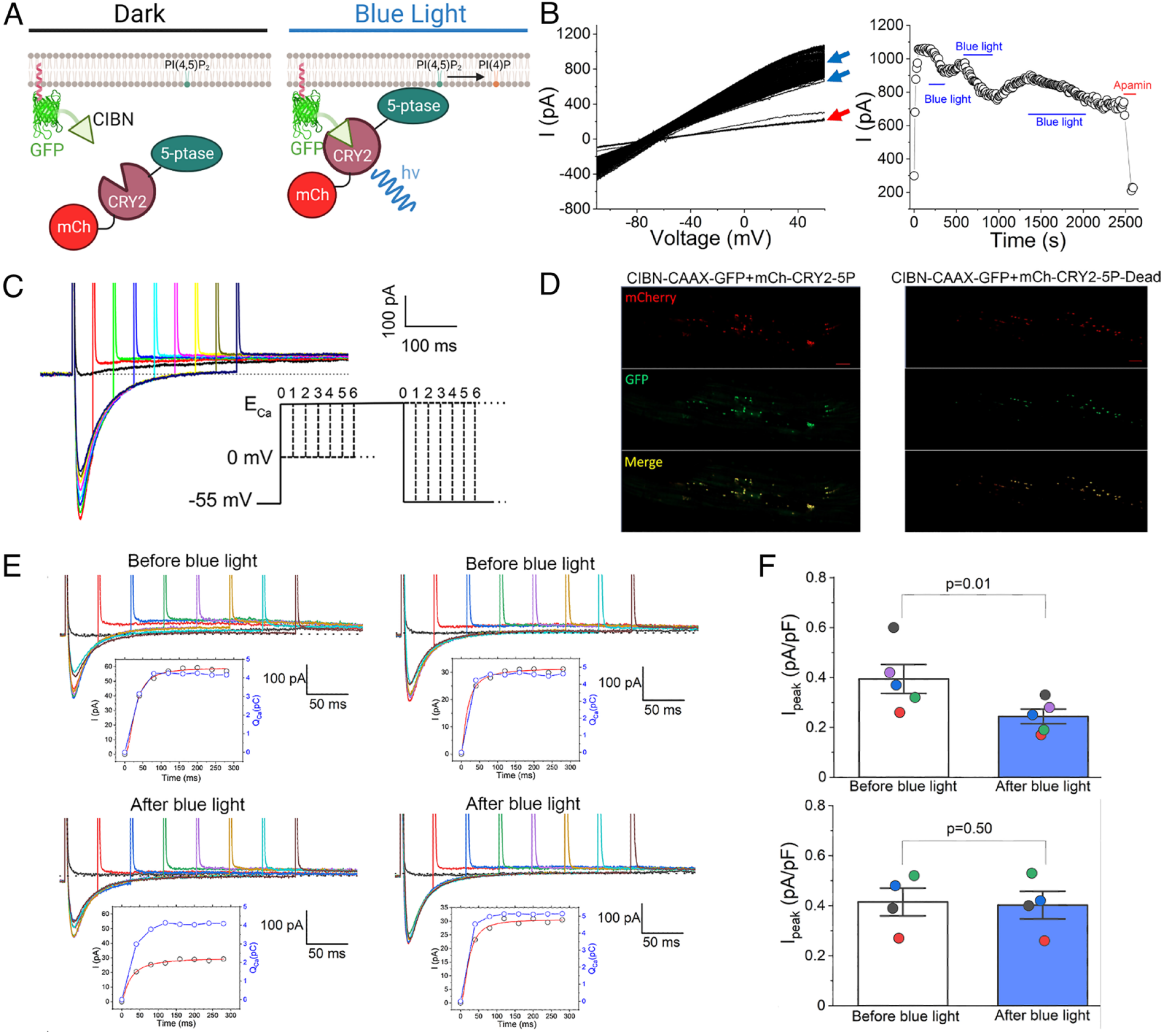
Regulation of *I*_K,Ca_ in cardiomyocytes using optogenetics. (*A*) Schematic illustrating the dimerization of the CRY2 and CIBN optogenetic constructs at the plasma membrane and dephosphorylation of PIP2 via inositol-5-phosphatase (5P) activity to produce phosphatidylinositol 4-phosphate (PIP). (*B*) *I*_K,Ca_ recorded in CHO cells using a voltage-ramp protocol prior to and during blue light exposure (blue arrow in the *Left panel* and black lines in the *Right panel*). (*C*) Current traces and the two-pulse voltage-clamp protocol used to isolate apamin-sensitive current activated by Ca^2+^ influx through L-type Ca^2+^ channels (LTCCs). The voltage-clamp protocol uses a prepulse to progressively induce Ca^2+^ influx via LTCCs from a holding potential of −55 mV, followed by a test pulse to monitor the apamin-sensitive *I*_K,Ca_. We recorded Ca^2+^ current (*I*_Ca_) for each cell to measure the reversal potential (*E*_Ca_). The test pulse was stepped to the observed *E*_Ca_ to minimize inward *I*_Ca_. Specific inhibitors for transient outward K^+^ current (*I*_to_), rapidly activating (*I*_Kr_) and slowly activating delayed rectifier K^+^ currents (*I*_Ks_), inward rectifier K^+^ currents (*I*_K1_), and Cl^−^ currents were applied. The instantaneous outward K^+^ currents progressively increased depending on the Ca^2+^ influx. (*D*) Rabbit ventricular myocytes were transfected with optogenetic constructs containing inositol-5-phosphatase (5P, *Left panel*) compared to control (inactive phosphatase, 5P-Dead, *Right panel*) using magnetic nanoparticles. (*E*) The activation kinetics of *I*_K,Ca_ was quantified compared to the total charge entered through LTCC during the prepulse (Q_Ca_ in pC). Current traces together with insets showing activation kinetics of *I*_K,Ca_ (red line) compared to the total charge entered through LTCCs during the prepulse (Q_Ca_ in pC, blue line), before and after the blue light for rabbit ventricular myocytes transfected with optogenetic constructs containing inositol-5-phosphatase (5P, *Left Panel*) compared to control (inactive phosphatase, 5P-Dead, *Right Panel*). (*F*) *I*_K,Ca_ was significantly blocked after blue light exposure compared to control cells. *P = 0.01.

We first tested the optogenetic constructs (Fig. 1*A*) by recording apamin-sensitive current (*I*_K,Ca_) from Chinese hamster ovary (CHO) cells expressing *hSK2* subunits using a voltage ramp protocol (120 to +60 mV from a holding potential of −55 mV). The biomolecular probes were previously developed (48) (Fig. 1*A*). The system is based upon dimerization between two proteins: i) the transcription factor CIBN is fused to a plasma membrane anchor, CAAX, and ii) *Arabidopsis thaliana* cryptochrome 2 (mCherry-CRY2) fused to a PIP2 5-phosphatase (5P). At rest the 5P is cytoplasmic but translocates to its plasma membrane CIBN-CAAX dimerization partner upon the application of blue light (458 nm), resulting in dephosphorylation and subsequent depletion of PIP2 within ms and can be observed within seconds (Fig. 1*A*).

*I*_K,Ca_ was recorded continuously before and after blue light exposure. Fig. 1B illustrates a significant reduction in *I*_K,Ca_ in CHO cells during blue light exposure (blue line) that recovered when the blue light was turned off. Apamin was applied at the end of the experiments to document apamin sensitivity.

Our results are consistent with the previous studies demonstrating that PIP2 depletion leads to a reduction in SK2 currents in the expression system (36). However, the regulatory roles of PIP2 on SK channels in cardiomyocytes remain unknown. To directly determine the regulation of SK channels by PIP2 in cardiomyocytes, a combination of functional analyses and optogenetics was utilized (Fig. 1 *C* and *D*). *I*_K,Ca_ was elicited using two-pulse protocols as previously described (18) (Fig. 1*C**)*. Rabbit ventricular myocytes were transfected with optogenetic constructs containing protein phosphatase (5P, *Left Panel*, Fig. 1*D*) compared to control (inactive phosphatase, 5P-Dead, *Right Panel*, Fig. 1*D*). Current traces together with insets showing activation kinetics of *I*_K,Ca_ (red line) compared to the total charge entered through L-type Ca^2+^ channels (LTCCs) during the prepulse (*Q*_Ca_ in pC, blue line) are shown before and after blue light exposure in Fig. 1*E*. Summary data show that *I*_K,Ca_ was significantly inhibited after blue light exposure compared to control cells (*P = 0.01, Fig. 1*F*) confirming a vital role of PIP2 in cardiac SK channel activation.

### The *h*SK2 Models Derived from Homology Modeling and MD Simulations.

Having established the critical roles of PIP2 in regulating *I_K,Ca_* in cardiomyocytes, we directly determined the atomistic mechanisms of how PIP2 works in concert with Ca^2+^-CaM in the SK channel activation. We first established homology *h*SK2 models using the cryo-EM structures of *h*SK4 channel complexed with CaM in closed, intermediate, and open states (PDB IDs: 6CNM, 6CNN, and 6CNO, respectively) (47).

**Fig. 2.**
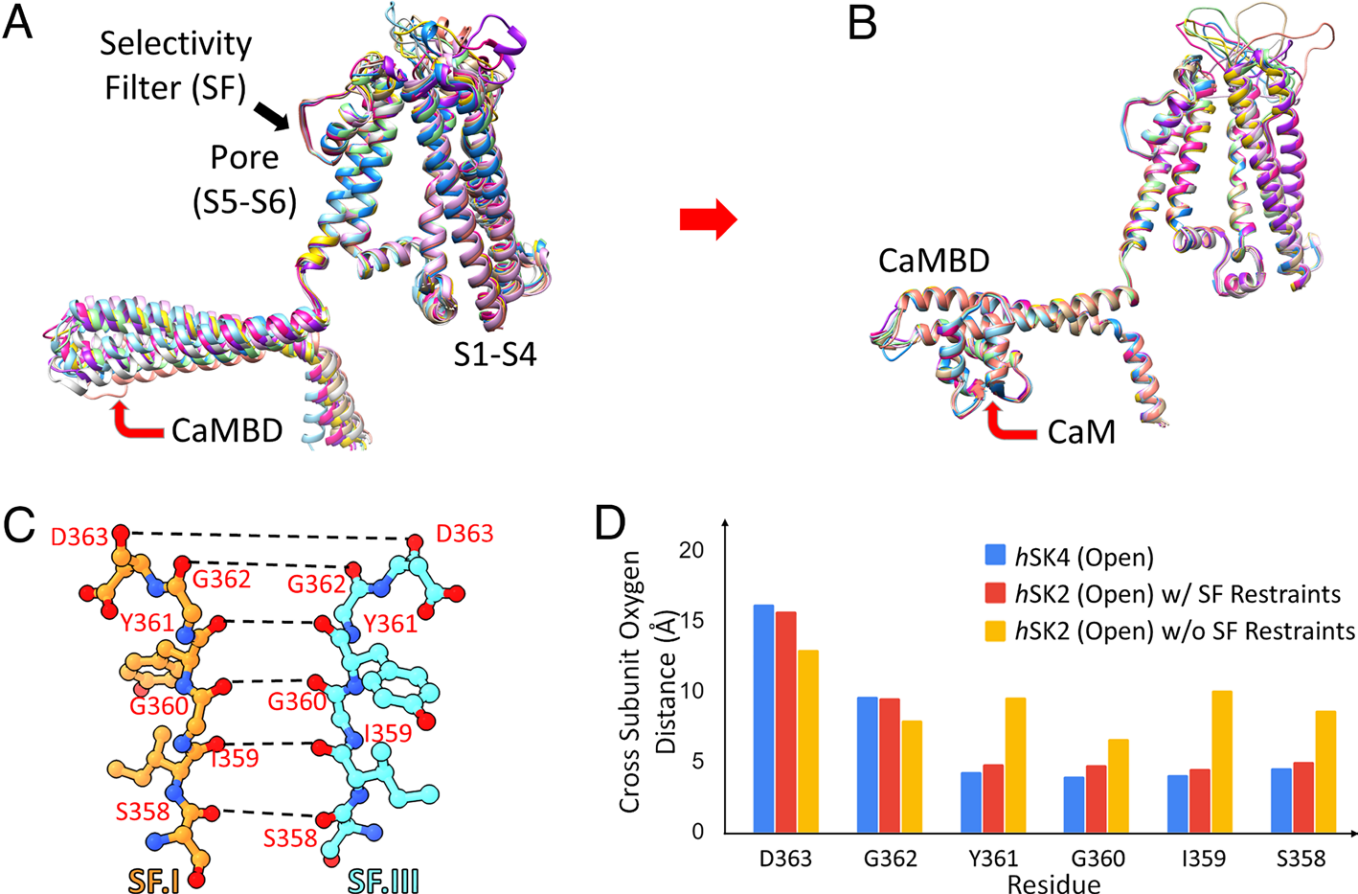
Homology models of *h*SK2–CaM complexes based on the open state of *h*SK4-CaM cryo-EM structure (PDB: 6CNO) using Rosetta structural modeling. (*A*) Top 10 homology models of the *h*SK2 channel without CaM show large movements in the CaMBD in the C terminus of *h*SK2 channels, due to the flexible loop connecting pore-lining transmembrane helix S6 to the CaMBD. (*B*) Inclusion of CaM in homology modeling shows convergence among the top 10 models for the CaMBD. (*C*) Selectivity filter (SF) of the *h*SK2 channel shown using ball-and-stick representation with the distances of backbone carbonyl oxygen atoms (red balls) shown as dashed black lines (amino acid residue numbering based on the *h*SK2 channel). Nitrogen atoms are shown as blue balls. (*D*) Cross-subunit backbone carbonyl oxygen distances for SF amino acid residues of the open state of *h*SK4 cryo-EM structure (blue bars), *h*SK2–CaM homology models with 100 kcal/mol/Å^2^ restraints being applied to the backbone C_α_ atoms (red bars), and *h*SK2–CaM homology models without (w/o) restraints (yellow bars).

We directly compared the sequence between *h*SK2 (UniProt ID: Q9H2S1) and the cryo-EM structure of *h*SK4 (PDB ID: 6CNM) used as the template (*SI Appendix*, Fig. S1). The sequence identity between the *h*SK4 and *h*SK2 structures is 46%, while the sequence similarity is 63%. We further compared the sequence for the CaM binding domain (CaMBD) in the C-terminal domain of the *h*SK2 channel (residues 412 to 488). The sequence identity between the two CaMBDs is 47%, with a sequence similarity of 68%. Since sequence identity is >30 to 40%, *h*SK4 structures represent sufficiently good templates for *h*SK2 structural models (49, 50). We employed the modified Rosetta Comparative Modeling (RosettaCM) protocol to generate *h*SK2 homology models (51–58), via two main steps: 1) cryo-EM map refinement and 2) homology modeling (*SI Appendix*, Fig. S2). Additional restraints were imposed during the homology modeling process to maintain the integrity of the selectivity filter (SF) and CaMBD. The absence of CaM in the models triggered large movements in the CaMBD due to the hinge loop connecting the S6 transmembrane segment to the CaMBD (Fig. 2*A*). In contrast, the inclusion of CaM led to convergence and improved agreement in the top *h*SK2–CaM models for the CaMBD (Fig. 2*B*). Applying restraints to the backbone C_α_ atoms (100 kcal/mol/Å^2^) in the *h*SK2–CaM homology modeling results in a stable SF as shown compared to the cryo-EM structures (Fig. 2 *C*–*D*).

**Fig. 3.**
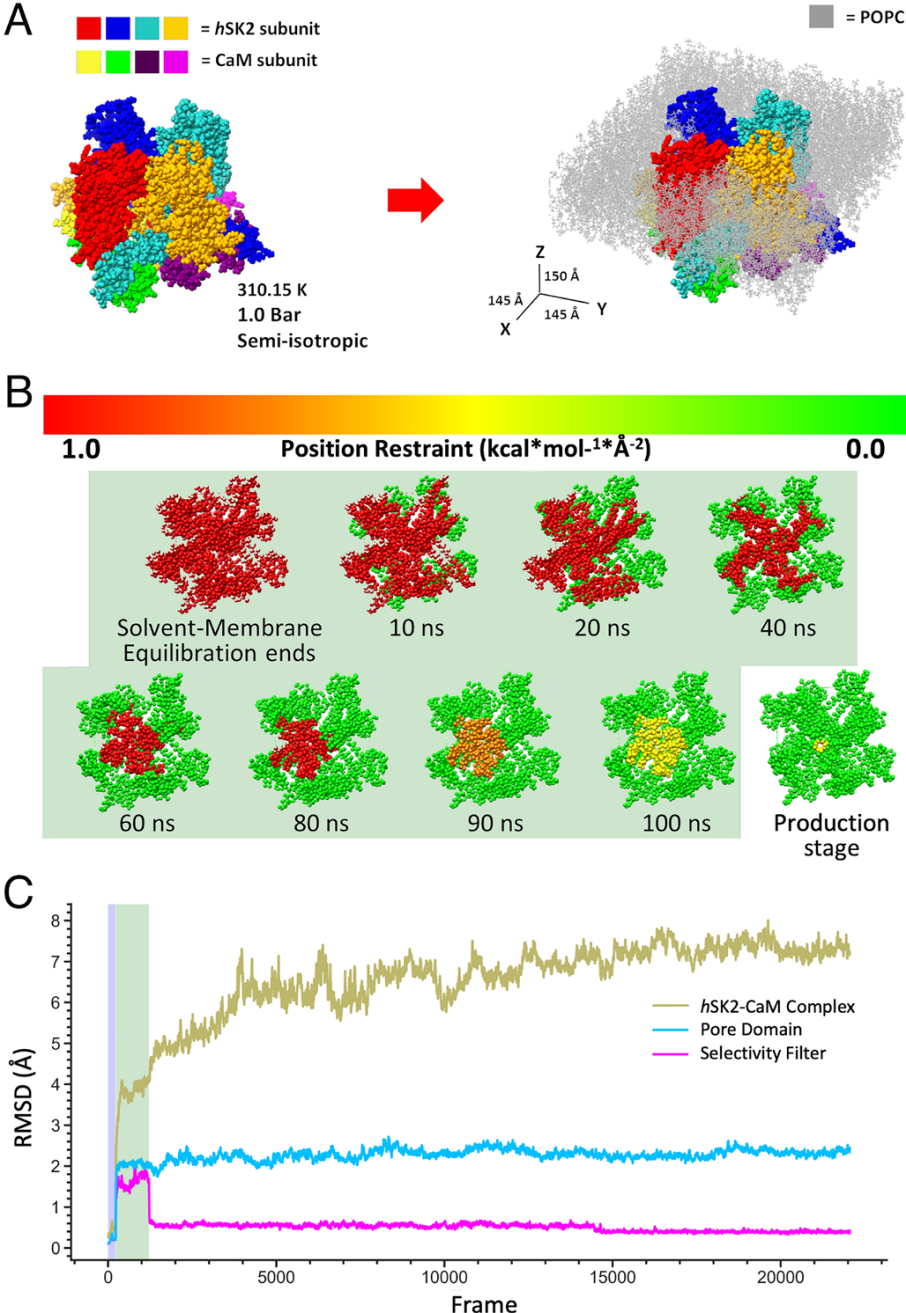
MD simulation parameters for a *h*SK2–CaM complex: (*A*) Visualization of the four subunits of *h*SK2–CaM complex before (*Left*) and after (*Right*) embedding into a POPC bilayer (light gray). PIP2 molecules are included in the lower bilayer leaflet only but are not visualized. MD simulation conditions and box size dimensions in *X*, *Y,* and *Z* are shown. (*B*) An extended equilibration protocol for 100 ns using Amber18 on the high-performance computer cluster (HPC) Expanse provides stable MD simulations by varying the location and strength of the restraints applied to the *h*SK2–CaM tetramer up to each time point indicated in ns and shaded in light green. It was followed by mostly unrestrained production run on Anton 2 supercomputer. (*C*) RMSD profiles for the backbone C_α_ *h*SK2–CaM tetramer and the selectivity filter (SF) during MD simulations equilibrated for 100 ns using Amber18 (light green shaded area corresponding to the shaded area in panel *B*), followed by production runs of up to 5,000 ns on Anton 2.

The *h*SK2–CaM complex models derived from homology modeling were embedded into 1-palmitoyl-2-oleoyl-sn-glycero-3-phosphocholine (POPC) lipid membrane solvated by 0.15 M KCl aqueous solution using CHARMM-GUI (Fig. 3*A*) and used in MD simulations as was performed in previous studies on similar biomolecular systems (59–61). *SI Appendix*, Table S1 summarizes 18 MD simulations on Anton 2 of *h*SK2–CaM complex in POPC membrane with or without PIP2 at 2.5, 5, and 10% (62–64). An extended equilibration protocol with gradually diminished restraints was performed on the high-performance computer (HPC) Expanse with Amber18 (Fig. 3*B*). Post-100 ns of equilibration, the simulations were transferred to Anton 2 for production runs, which extended up to 5 μs (Fig. 3*C*). To ensure system stability, position restraints were applied to the *h*SK2–CaM complex or its components at each step of the equilibration MD runs, and weak restraints on *h*SK2 SF backbone atoms were maintained throughout production runs as well (Fig. 3*B*). The MD simulation trajectories were analyzed via root mean square deviation (RMSD) calculations (Fig. 3*C*), demonstrating stable *h*SK2–CaM complex as well as the SF throughout the 5-μs MD simulations.

### Pore Mapping Using HOLE Analyses and Validation of the Pore Conductivity.

**Fig. 4.**
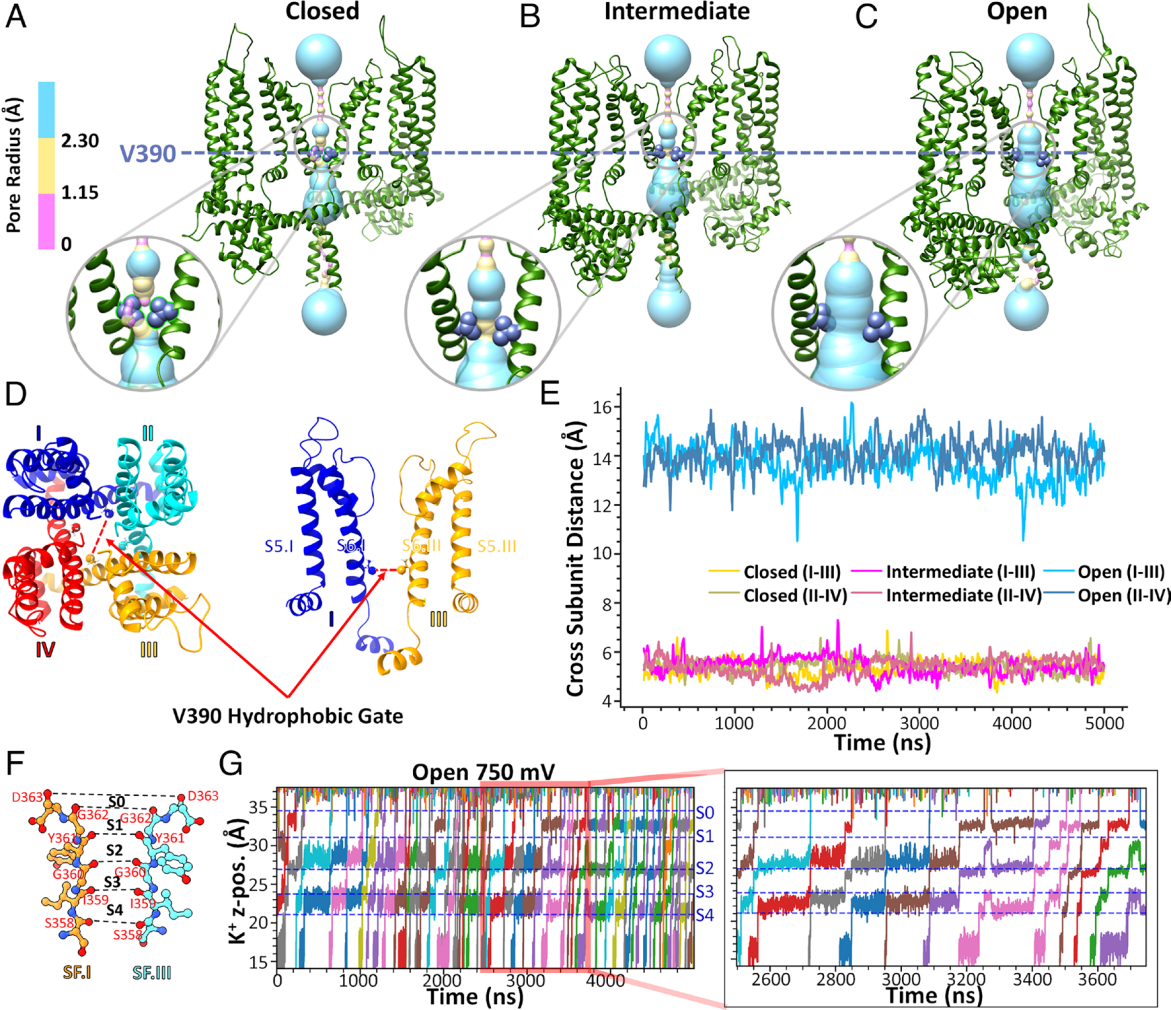
Intracellular hydrophobic gate V390 residue across-subunit distances and K^+^ ion conduction for homology models of the *h*SK2–CaM tetramer. (*A*–*C*) Location of the V390 residue in the S6 transmembrane helix of the *h*SK2–CaM tetramer in closed, intermediate, and open conformational states. Pore dimensions were computed using the HOLE program and are shown using surface representation with coloring corresponding to the pore radii. Insets in A-C are zoomed in images. (*D* and *E*) Quantification of the across-subunit distances between backbone C_α_ atoms of V390 residues (open = blue, intermediate = magenta, and closed = yellow). (*F* and *G*) Illustration of ion conduction in the open conductive homology model of *h*SK2–CaM tetramer. The time series of K^+^ ion *z*-positions (K^+^ z-pos.) during 5-μs MD simulations with an applied voltage of 750 mV is depicted as they pass through the selectivity filter (SF). Five distinct positions of K^+^ in the SF, sites S0 to S4, defined by the backbone carbonyl oxygen atoms (colored in red) of SF amino acid residues (S358 to D363) have been labeled and separated by horizontal dashed lines. The inset on the right panel shows K^+^ ion conduction at an expanded time scale.

To further validate the homology models for *h*SK2–CaM complexes that we have generated, central ion channel pore mapping using the HOLE program (65) (Fig. 4) was performed to confirm the closed, intermediate, and open states of the homology models. Fig. 4 *A*–*C* highlights the radius profiles across the entire channel pore and intracellular hydrophobic gate region at the V390 residues in the pore-lining S6 helices of the *h*SK2–CaM tetramer, in closed, intermediate, and open states (only *h*SK2 pore domain is shown for clarity). Intracellular hydrophobic gates at the V390 residues were observed as the narrowest region in the lower part of the central pore of the *h*SK2 channels and was measured as the distance between backbone C_α_ atoms of opposite subunits, i.e., an across-subunit distance. Time series profiles of the across-subunit distances between V390 residues (Fig. 4*D*) are shown in Fig. 4*E* with consistent distances of ~6 Å in closed and intermediate state models and ~14 Å in the open-state models throughout the 5-μs-long MD simulations.

We further validated the conductivity of the SK2–CaM complex homology models in the open state. We meticulously tracked the trajectory of K^+^ ions over a 5-μs MD simulation (Fig. 4 *F*–*G* and Movie S1). After applying 750 mV transmembrane voltage using an applied electric field approach (66) with 150 mM of K^+^ ions in aqueous solution and 4 K^+^ ions seeded in the central pore and 3 K^+^ ions in the SF, we observed multiple K^+^ permeation events corresponding to the single channel conductance of ~2.47 pS, which is in close agreement to the experimental value for the SK2 channels (7).

### Tracking the Trajectory of PIP2 Binding to the *h*SK2–CaM Complex During MD Simulations.

**Fig. 5.**
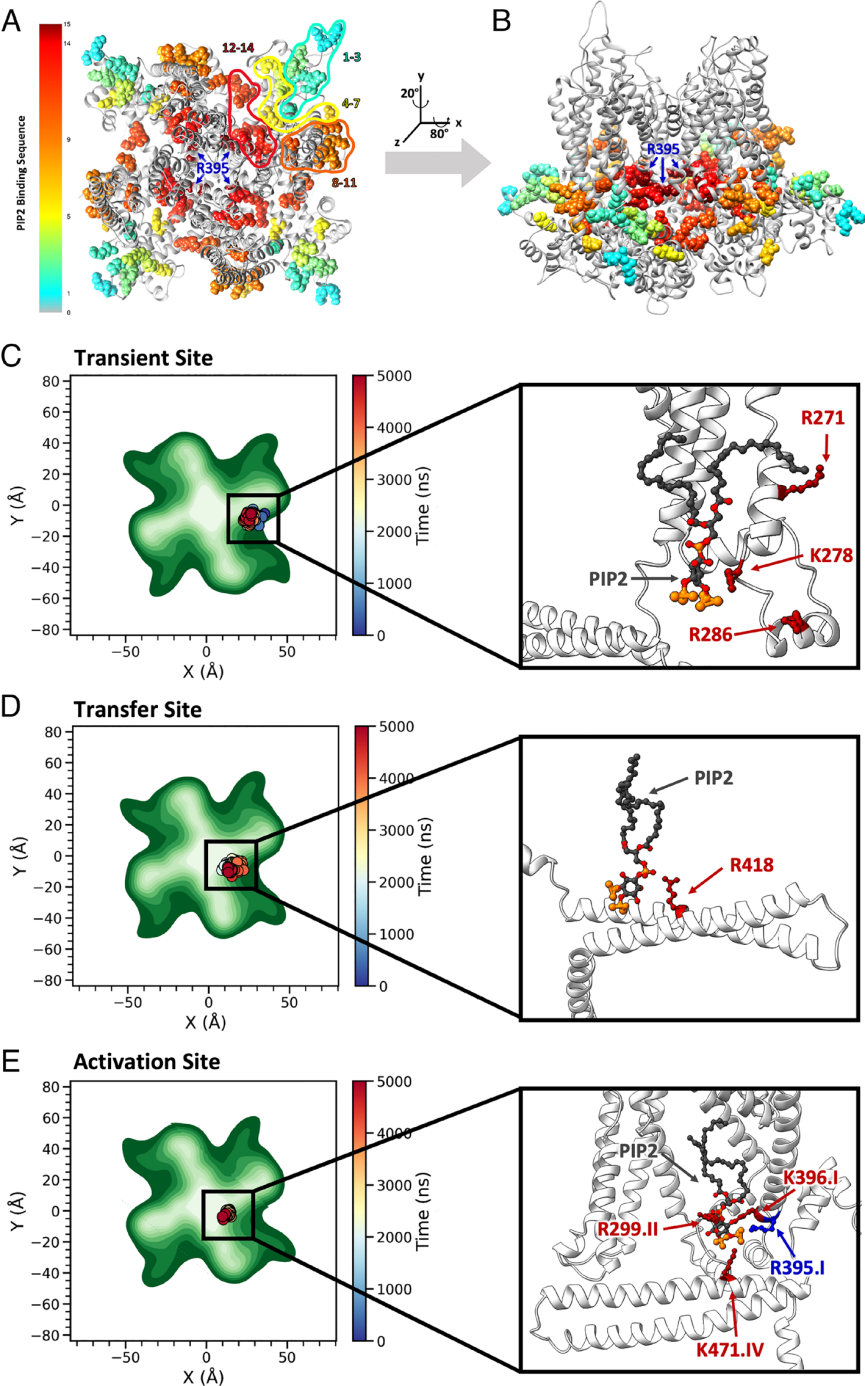
Tracking of PIP2 in the membrane *XY* plane over time identifies two PIP2 binding pockets, denoted transient and activation binding sites. (*A* and *B*) Movement of PIP2 molecules into the binding pockets of the *h*SK2–CaM homology model in the intermediate state during MD simulations. The color scale provides time progression over the 5-μs MD simulations from cyan to green, yellow, orange, red, and dark brown. The *h*SK2–CaM complex is shown with top (from the extracellular side) and side views in panels *A* and *B*, respectively. (*C*–*E, Left Panels*) Top views of the *h*SK2 channel as in A. PIP2 molecule 1, 2, and 3 locations at the transient, transfer, and activation binding sites during 5 μs of the MD simulations. PIP2 location is shown by colored dots corresponding to the simulation time with the corresponding time scale on the right, whereas *h*SK2–CaM complex atomic density is shown in different shades of green with darker colors corresponding to higher density. (*C*) PIP2 molecule 1 at the transient binding site, which includes amino acid residues R271, K278, and R286. (*D*) Transfer of PIP2 molecule 2 from the transient to the activation binding site. (*E*) PIP2 molecule 3 at the activation binding site, which includes residues R299, R395, K396, and K471. (*C–E, Right Panels*) Magnified side view of the binding sites of PIP2 on *h*SK2 channel (side view as in Panel *B*) to further illustrate amino acid residues crucial for PIP2 binding.

PIP2 molecules were randomly placed in the lower leaflet of the lipid bilayers in the range of 2.5 to 10% during MD simulations to obtain unbiased PIP2 binding sites and the dynamics of PIP2 binding. The concentrations of PIP2 were chosen based on recent estimations of PIP2 in the lower leaflet of the lipid bilayer that can be as high as 2 to 5% (67). Indeed, MD simulations provided valuable insights into how PIP2 binds to the basic residues of the SK2 channels. Using the concepts of ligand binding as a foundation, we designed an analysis protocol focusing on salt bridges formed between the *h*SK2 channel and PIP2, via a 5.5 Å cutoff from the center of the charges. The analyses enabled the tracking of PIP2 molecules as they laterally move in the lipid membrane traversing along different amino acid residues. Fig. 5 *A*–*B* and *SI Appendix*, Fig. S3 show the movement of PIP2 into the binding pockets of the *h*SK2–CaM complex during 5-μs MD simulations from the membrane periphery. PIP2 initially reaches and binds to the residues depicted in cyan in Fig. 5 *A*–*B*, sequentially transitioning to other binding sites depicted in green, yellow, orange, red, and finally, dark red during 5-μs MD simulations. Two main PIP2 binding pockets were identified, denoted as “transient site” and “activation site” with translocation from transient to activation sites via the “transfer” amino acid residue R418 (Fig. 5 *C*–*E*).

### Formation of salt bridges between PIP2 and amino acid residues from the *h*SK2–CaM tetramer.

MD simulations provide significant insights into how binding of PIP2 within the “activation site” results in *h*SK2 channel activation. We determined whether PIP2 binding would disrupt any salt bridges that stabilize different intrasubunit transmembrane helices (S1-S6, CaMBD helices A-C (HA-HC)) or intersubunit interactions. *SI Appendix*, Fig. S4 summarizes key intra- and intersubunits salt bridges in the *h*SK2-CaM structure. Remarkably, based on these analyses, we only found one salt bridge that was routinely disrupted by PIP2 binding, specifically, the R395:E398 salt bridge from S6 transmembrane segments of two adjacent subunits of the *h*SK2 channel, located close to the hydrophobic gate S6 residue V390.

**Fig. 6.**
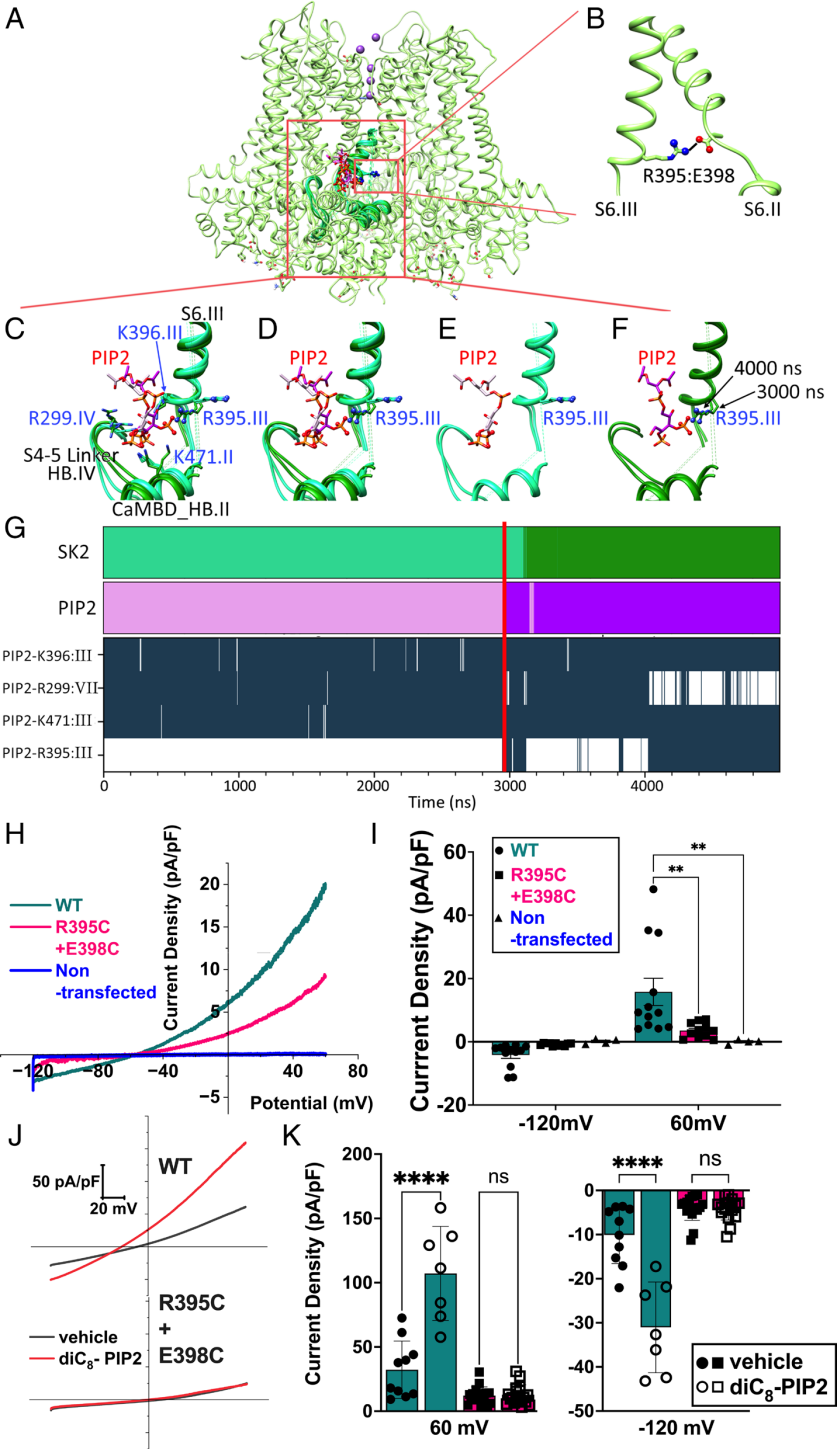
Formation of salt bridges between PIP2 and amino acid residues from the *h*SK2–CaM tetramer and their disruption by mutagenesis experiments. (*A*) *h*SK2–CaM complex (shown as green ribbons with 70% transparency) to highlight the location of PIP2 binding shown as a wireframe. Only two subunits are shown for clarity. The red box highlights the region of zoom-in panels in *B*–*F*. (*B*) Zoom-in view of the intersubunit salt bridge between R395 of subunit III and E398 of subunit II located on S6 helices shown as ribbons. (*C*–*F*) PIP2 carbon atoms are shown in pink and *h*SK2 backbone is shown in green. Phosphorus (orange), oxygen (red), and nitrogen (blue) atoms involved in PIP2:R395 salt bridges are shown in ball-and-stick representation. Representative poses of *h*SK2–CaM from clustering of MD simulation data that are able to form the PIP2:R395 salt bridge are shown in darker shades and poses unable to form this salt bridge are shown in lighter shades. (*C*) Poses with all major amino acid residues forming salt bridges with PIP2 in the activation site, R299, R395, K396, and K471. (*D*) R395 side chain orientations shown to exemplify differences when it forms a salt bridge with E398 vs. PIP2. (*E*) Only *h*SK2–CaM poses that do not form the PIP2:R395 salt bridge. (*F*) Only *h*SK2–CaM poses that do form the PIP2:R395 salt bridge. (*G*) Two top graphs show clustering of MD simulation results based on simulation time showing *h*SK2–CaM (SK2) and PIP2 poses, which are able to form the PIP2:R395 salt bridge in darker shades of green and pink, respectively. The four lower graphs show time series of salt bridge formation (dark-blue) and breaking (white) between several *h*SK2–CaM residues and PIP2 using a 3.6 Å cutoff. The red vertical line across all the graphs shows time point at which R395:PIP2 salt bridge was first detected. (*H*) Apamin-sensitive currents from *h*SK2-WT compared to tandem constructs containing R395C and E398C mutations in *h*SK2 subunits 1 and 2, respectively. Nontransfected HEK293 cells do not exhibit appreciable apamin-sensitive currents under our recording conditions (blue trace). (*I*) Summary data showing the apamin-sensitive current density at −120 and +60 mV, respectively. (*J*) Representative current traces showing responses of WT compared to the mutant constructs to diC_8_-PIP2 in HEK293 cells with diC_8_-PIP2 (red traces) or vehicle alone (black traces) in the intracellular solution. (*K*) Summary data demonstrating *h*SK2 current density from WT compared to the mutant constructs. *h*SK2-WT current density was significantly enhanced by diC_8_-PIP2, while the mutant construct failed to respond to PIP2.

The Fig. 6 *A* and *B* highlight the salt bridge formation between residues R395 and E398 of two adjacent *h*SK2 subunits, Fig. 6 *C*–*F* focuses on clustering MD simulation poses with and without PIP2:R395 salt bridge, showing PIP2 interactions with other basic *h*SK2 residues, whereas Fig. 6*G* demonstrates dynamics of salt bridges between PIP2 and other amino acid residues. In Fig. 6 *C*–*F* and top panels in Fig. 6*G*, representative poses from clustering of *h*SK2–CaM MD simulations that are able to form the PIP2:R395 salt bridge are shown in darker shades of pink and green for PIP2 and *h*SK2, respectively, and poses unable to form this salt bridge are shown in lighter shades. As shown in Fig. 6 *C*–*G*, *h*SK2 amino acid residues forming salt bridges with PIP2 in the “activation site” include R299, R395, K396, and K471. The formation of the PIP2:R395 salt bridge correlates with breaking of the PIP2:R299 salt bridge, whereas PIP2 interactions with K396 and K471 are unaffected as shown in Fig. 6*G*. The residue R395 shows a significant change in the side chain orientation when it forms a salt bridge with either E398 or PIP2, as exemplified in Fig. 6 *E*–*F*.

### Additional Evidence for the Role of the Salt Bridge Formation Between R395:PIP2 From Solvent-Accessible Surface Area (SASA) Analyses.

SASA analyses were performed to quantify the solvent exposure of each amino acid in the “transient”, “transfer”, and “activation” PIP2 binding sites from the closed, intermediate, and open states. There were no significant differences in the SASA over time or averaged SASA for each amino acid residue within the same *h*SK2 conformational state in the POPC or POPC/PIP2 membrane if a PIP2 molecule did not directly interact with that amino acid residue. Therefore, we directly compared the SASA for each amino acid residue from the four subunits for each frame based on whether the residue is bound *vs.* unbound by PIP2 (the cutoff distance between a charged oxygen bound to a phosphorus on the PIP2 head group and a charged side chain nitrogen of basic amino acids of <4Å is defined as bound and >4Å is defined as unbound) and quantified changes in the SASA as the residue establishes the new salt bridge with PIP2 (*SI Appendix*, Fig. S5).

The *SI Appendix*, Fig. S5 shows the summary data for SASA of individual amino acid residues in each MD simulation and the average (shaded bar) of all simulation frames. SASA has been shown to provide a measure of the thermodynamic parameters of hydration of proteins, where the free energy of hydration is composed of the additive contributions of various functional groups and proportional to SASA (68). A larger SD of SASA may imply a greater variance in the environment, whether from the side chain movement, the surrounding residues or the lipid environment. Indeed, upon binding of PIP2 to an amino acid residue, we observed a decrease in the variance of the SASA values represented by SD and shown as error bars in *SI Appendix*, Fig. S5. Consistently, there is also a decrease in the number of side chain rotamers upon PIP2 binding.

We quantified simultaneous SASA and the salt bridge formation for the amino acid residue R395 in the intermediate state of the *h*SK2–CaM. With a transient salt bridge formation between the R395 residue and PIP2 (e.g., at 3,000 ns of MD simulation, the yellow box in *SI Appendix*, Fig. S5 *D* *Bottom* panel), there is no distinguishing feature in SASA corresponding to the transient salt bridge formation despite an obvious rotamer change of this amino acid residue in the MD clustering results (Fig. 6 *C*–*F*). In contrast, there is a simultaneous increase in the SASA with a significant decrease in the variance, occurring with stronger salt bridge formation (e.g., at 4,000 ns, the red box in *SI Appendix*, Fig. S5 *D* *Bottom* panel). Indeed, two distinct clusters of rotamers for the R395 residue exist in the bound state by PIP2 (between 3,000 to 4,000 and 4,000 to 5,000 ns for the first and second clusters, respectively for the MD simulation described, Fig. 6*F*). These findings suggest that the R395 residue may transition through an intermediate state between forming a salt bridge with residue E398 and then PIP2. Finally, we observed simultaneous changes in the SASA values of the adjacent residue K396 when residue R395 forms multiple salt bridges with PIP2 at ~4,000 ns of the MD simulation, most likely due to the exclusion of the residue K396 SASA by the R395 rotamers (the red box in *SI Appendix*, Fig. S5 *E* *Bottom* panel). Moreover, these observations were reproduced in another independent MD simulation using the same starting structure.

The SASA values for amino acid residues K278, R299, K396, R418, and K471 that are not bound by PIP2 are similar among all the *h*SK2–CaM conformational states (*SI Appendix*, Fig. S5 *Top* panels). Consistently, there are no significant differences in the salt bridge or other nonbonded interactions among these amino acid residues with surrounding amino acid residues in the different *h*SK2–CaM conformational states. In contrast, amino acid residue R395 exhibits a considerable increase in SASA for the open state (*SI Appendix*, Fig. S5 *D* *Top* panel), suggesting that this amino acid residue plays a key role in the transition from intermediate to open state. The salt bridge R395:E398 disruption in the open state further supports this notion.

We observed significant changes in the SASA values between PIP2 unbound and bound for amino acid residues K278, R299, R395, and R418, with the most extensive changes for the residue K278 in the closed state and the residues R299, R395, and R418 in the intermediate state (*SI Appendix*, Fig. S5 *A*–*D* *Top* panels). Amino acid residues K396 and K471 exhibit very little change in SASA values upon PIP2 binding. Consistently, these residues exhibit no significant rotamer changes between PIP2 bound and unbound states, correlating well with no significant changes in SASA over time between those states.

### Changes in Cytoplasmic Gate Distance with PIP2 Binding.

We directly analyzed the effects of PIP2 binding on the across-subunit diameter of the cytoplasmic hydrophobic gate at the V390 residues of the pore-lining S6 helices. The diameter of the cytoplasmic gate distance was determined between CG (C_γ_) side-chain atoms of amino acid residue V390 of opposite subunits: I-III and II-IV. There is a decrease in R395:E398 salt bridge formation as the intracellular gate diameter increases (*SI Appendix*, Fig. S6).

*SI Appendix*, Fig. S7 *A* and *B* shows asymmetry of the gate distances between subunits I to III (Panel **A**) and II to IV (Panel **B**), upon PIP2 binding within one of the unbiased 5-microsecond-long MD simulations (same simulation as was analyzed in *SI Appendix*, Fig. S6*D*), with PIP2 initially bound to residue K396 in the *“*activation site*”*, transitioned into weakly bound to the amino acid residue R395, then strongly bound to the amino acid R395 in subunit III. The geometric average distances were computed to account for the asymmetry and did not show significant differences for the 3 time segments shown in **A–B** (*SI Appendix*, Fig. S7*C*). However, there was an increase in the geometric average distances with PIP2 binding compared to control MD simulations with no PIP2 present (compare panels **C** and **F**). The data were further summarized in *SI Appendix*, Fig. S7*G* and Table S2, demonstrating an increase in the cytoplasmic gate distances upon PIP2 binding.

### The R395C-E398C Tandem Mutant *h*SK2 Channel Fails to be Activated by PIP2.

In order to test the prediction of the critical roles of R395:E398 salt bridge on PIP2 regulation of the *h*SK2 channel, we generated a tandem construct of two *h*SK2 subunits containing R395C mutation in subunit 1 and E398C mutation in subunit 2 (Fig. 6 *H*–*K*). The results showed that compared to the wild-type (WT) constructs, the dual mutations in the tandem *h*SK2 subunits significantly reduced the current amplitude (Fig. 6 *H*–*I*). Nontransfected HEK293 cells exhibit no apamin-sensitive currents (Fig. 6 *H*–*I*). To test the prediction of the critical roles of R395 and E398 residues in PIP2 regulation of *h*SK2 channels, we applied PIP2 derivative diC_8_-PIP2 to the intracellular side via patch pipettes. In the presence of diC_8_-PIP2, there was a significant increase of the currents only in the *h*SK2-WT construct. However, the mutant construct failed to generate any observable responses to diC8-PIP2 (Fig. 6 *J*–*K*).

### Proposed Structural Mechanism of the PIP2-Induced Activation of SK2 Channels.

**Fig. 7.**
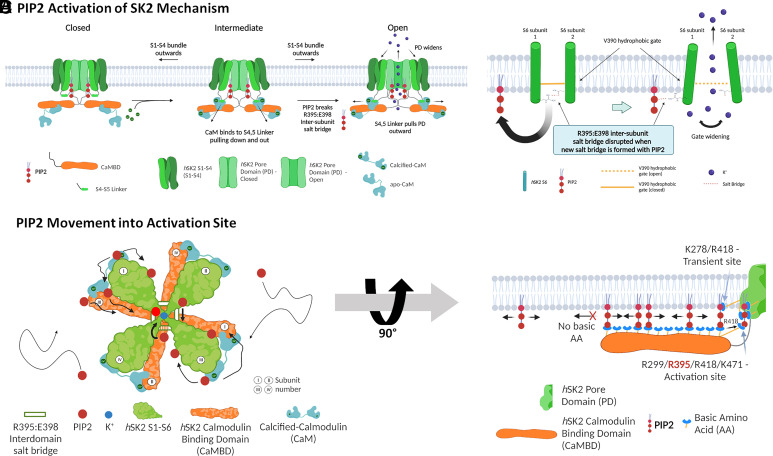
Diagrams depicting how PIP2 works in concert with Ca^2+^ and calmodulin (CaM) in the proposed activation mechanism of the SK2 channel. (*A*) The conformational state transition mechanism of the *h*SK2–CaM complex. (*B*) The widening of the SK2 intracellular gate through the disruption of the cross-subunit R395:E398 salt bridge by PIP2. (*C*) The movement of PIP2 into the SK2 activation site. (*D*) Depiction of the conveyor belt-like movement of PIP2 by binding to multiple basic residues in the CaMBD of SK2 channels.

Fig. 7 *A*–*B* summarizes our proposed atomistic structural and dynamic mechanisms of how PIP2 works in concert with CaM to activate the *h*SK2 channel. *h*SK2–CaM complexes are schematically depicted in closed, intermediate, and open states (only two subunits are shown for clarity). In the closed state, CaMBD (shown in brown) in the C termini of the *h*SK2 channel interacts with C-lobes of apo-CaM. Upon binding of Ca^2+^ to CaM in the intermediate state, the calcified CaM assumes an extended state, leading to the interactions of the N-lobe of CaM with the S4-S5 linker, pulling the linker down and out, enabling the binding of PIP2 to residue R395 in the intermediate state. Interactions of PIP2 with residue R395 result in the disruption of the R395:E398 salt bridge and the lowering of the energy barrier to transition to the open state.

Fig. 7 *C*–*D* illustrates multiple PIP2 molecules that reside within the inner leaflet of the membrane in the *h*SK2–CaM intermediate state with Ca^2+^ bound to the CaM of the *h*SK2 channel. PIP2 molecules were observed in the MD simulations to traverse sequentially through the basic amino acid residues in CaMBD, much like objects moving on a conveyor belt. Upon binding to residue R395, PIP2 disrupts the R395:E398 salt bridge between the two adjacent *h*SK2–CaM subunits (Movies S2 and S3). The disruption of the R395:E398 salt bridge triggers the expansion of the channel’s pore gate, facilitating the passage of K^+^ through the channel.

## Discussion

In the current study, we deciphered molecular mechanisms of PIP2 regulation of apamin-sensitive SK currents in cardiomyocytes, using optogenetics and magnetic nanoparticles, combined with functional and structural analyses. Furthermore, homology modeling combined with long unbiased all-atom MD simulations provide insights into the atomistic structural and dynamic mechanisms of how PIP2 works in concert with Ca^2+^-CaM to activate the *h*SK2 channel.

Previous studies have characterized PIP2 binding sites, however, the atomistic mechanisms and dynamics of *h*SK2 channel activation by PIP2 remain incompletely understood. Therefore, the goal of the computational study was to identify in an unbiased manner all key PIP2 binding sites, with random initial placement of PIP2 in the lipid membrane in the MD simulations. To this end, our computational study affords evidence for the critical role of the amino acid residue R395 in the S6 transmembrane segment, which is localized close to the cytoplasmic hydrophobic gate. This residue forms a salt bridge with residue E398 in the S6 transmembrane segment from the adjacent subunit. Both R395 and E398 are conserved in all known isoforms of SK channels. Specifically, our findings suggest that binding of PIP2 to the residue R395 results in the disruption of the R395:E398 salt bridge, an increase in the flexibility of the transmembrane segment S6, and the activation of the channel. More importantly, our findings serve as a prospective platform for future testing of structural-based drug designs for therapeutic inhibitors and activators of the SK channel family.

### Physiological Significance of PIP2 Regulation of Cardiac SK Channels.

SK channels serve to integrate the beat-to-beat rises in the intracellular Ca^2+^ with membrane potentials and hence ionic fluxes across the cell membrane of cardiomyocytes. The regulation of SK channels plays critical roles in cardiac excitability and arrhythmogenesis. Regulation of cardiac ion channels by PIP2 represents a key mechanism for the lipid regulation of cardiac function. PIP2 regulates multiple cardiac ion channels (40–44), therefore, PIP2 may play critical roles in arrhythmia initiation and maintenance (45, 46). Moreover, PIP2 is dysregulated in HF from the sustained phospholipase C activation, mediated via G protein-coupled receptors, which may significantly affect cardiac ion channel activities. Moreover, SK channels are the only known K^+^ channels up-regulated in HF (21, 33–35). Therefore, in-depth insights into the dynamic regulation of cardiac SK channels by PIP2 are critical for future development of therapeutic strategies for cardiac arrhythmias.

### Alternative Mechanisms of SK Channel Activation by PIP2.

Several prior studies have provided critical insights into the interactions of PIP2 with SK channels. One previous elegant study generated a crystal structure containing only the CaMBD of the *h*SK2 channel, CaM, and PIP2 (36). In contrast, our current study takes advantage of the cryo-EM structures of S1–S6 transmembrane helices and the CaMBD of a homologous ion channel *h*SK4 complexed with CaM, which we used to build atomistic structural models using Rosetta. As such, our current models are only missing the N-terminal and C-terminal flexible tail regions of the channel. Based on our current models, we observed that the previously generated crystal structure of CaMBD with bound CaM (36) displayed an orientation that would disrupt the full structure of an *h*SK2 subunit. In addition, mutations in the S6-CaMBD linker region, K402-K405, suggested by the previous study to directly disrupt PIP2 binding (36), are predicted in our model to alter multiple salt bridge networks, that may result in changes in the overall conformation of the channel.

The previously reported crystal structure also implicated a different binding pocket for PIP2, which involves the S6-CaMBD flexible linker from *h*SK2 and residues R75 and K78 from CaM (36). In our current model, these amino acid residues R75 and K78 are also involved in an intricate salt bridge network with the *h*SK2 subunit. The previous study further demonstrated that phosphorylation of residue T80 of CaM or the T80D phospho-mimetic mutant CaM prevents the activation of the *h*SK2 channel. The authors suggested that PIP2 serves as a source of phosphate for the phosphorylation of the T80 residue, and the coordinated *h*SK2–CaM binding pocket for PIP2 prevents the phosphorylation of the T80 residue, thus allowing the activation of SK2 channel (36).

A follow-up study from the same group of investigators (37) included NS309, an SK channel activator, in the overall crystal structure. They noted that NS309 does not increase the Ca^2+^-bound CaM in the CaMBD–CaM complex, suggesting that NS309 may enhance *h*SK2 channel activation after Ca^2+^ binding to CaM in the intermediate state by potentially increasing PIP2 affinity. While their crystal structure only contains the C-terminal domain of the *h*SK2 channel, the investigators proposed a PIP2 binding site that is relatively close to *h*SK2 residue K418, which transfers PIP2 from the “transient” binding site to the “activation” binding site in our MD simulation study (Fig. 7*D*). Additional experiments and MD simulations will be highly informative to investigate how NS309 interacts with PIP2.

A more recent study used the cryo-EM structure of the *h*SK4-CaM complex in the intermediate state (PDB: 6CNN) to design a blocker for the putative *h*SK4-CaM coordinated binding of PIP2, with a series of mutations and molecular docking simulations (38). The docking of PIP2 to *h*SK4-CaM complex identified residues R180, R191, R352, R355, and R359 of *h*SK4 and R75 of CaM to be involved in PIP2 binding sites. These *h*SK4 residues correspond to R286, N297, R463, K466, and R470 in the *h*SK2 channel, respectively. Single point mutations of *h*SK4 at these residues (R180A, R191A, R352Q, R355G, and R359G) and K76A mutation in CaM disrupted the activation of *h*SK4 channel by PIP2 (38). The corresponding *h*SK2 amino acid residues are involved in the formation of salt bridges in our MD simulations that either directly involve CaM, an adjacent *h*SK2 subunit, or may provide stability to the S4-S5 linker that binds CaM. Since the docking method used in that study (38) does not constrain the PIP2 molecule to be located in the lipid membrane, the findings differ in the exact binding residues with our study, but only by 1 to 3 residues. In addition, the designed inhibitor is specific to the *h*SK4 channel due to the π-stacking of the inhibitor to the *h*SK4 residue H192, which is not conserved in SK1-3 channels.

Our study identified the R299 residue on the *h*SK2 channel to be the first amino acid residue that PIP2 binds as it transitions into the activation binding site. However, the R299 residue is only conserved in SK1-3 but not SK4 channel. Deviations in atomistic mechanisms by which PIP2 regulates SK1-3 and SK4 channels may contribute to the activation kinetic differences between the channels.

### MD Simulations with PIP2 Embedded in the Membrane.

Prior studies have provided insights into PIP2 binding on the SK channels, however, the dynamic and structural atomistic mechanisms of how PIP2 assists in the transition of the SK channel into an open state remains unknown. In addition, we aimed to explore more physiologically relevant conditions where PIP2 molecules are embedded in the inner leaflet of the lipid bilayer and not floating free in the cytoplasm. Surprisingly, in our multimicrosecond-long MD simulations of PIP2 we detected no binding to previously predicted CaM binding residues (R75/K78). However, PIP2 did bind CaM at residue K116 while it trafficked along the CaMBD to the “transient site”. Therefore, a global search for binding sites may be more comprehensive and informative as local docking can be biased by the initial placement of a ligand.

In addition, conventional analyses of MD simulations for ligand binding to the receptors that include dwell time, disassociation kinetics, or percentages of time with ligand binding may not apply due to the relatively slow kinetics in which PIP2 traverses in the membrane compared to a free-floating ligand. Using the concepts of ligand binding as a foundation, we designed an analysis protocol focusing on salt bridges between the *h*SK2–CaM complex and PIP2. The analyses enable the tracking of PIP2 molecules as they traverse along different amino acid residues, while still located within the membrane. We then directly determined whether PIP2 binding would disrupt salt bridges that stabilize different transmembrane helices (S1 to S6) or adjacent *h*SK2 subunits. Remarkably, based on these analyses, we only found one salt bridge that was routinely disrupted by PIP2 binding, specifically, the cross-subunit R395:E398 (Movies S2 and S3), located close to the hydrophobic gate V390 residue in the S6 transmembrane domain.

Our analyses support the findings that PIP2 can only bind the R395 residue in the intermediate conformational state of *h*SK2–CaM. The R395:E398 salt bridge is observed to be present in all states but the frequency of formation is highest in the closed state and lowest in the open and conducting state where the gate is at the largest distance between subunits. The SASA for residue R395 in the intermediate state significantly increases upon PIP2 binding. Consistently, the SASA for residue R395 also exhibits a substantial increase in the open state. The salt bridge R395:E398 disruption in the open state further supports the notion that the R395:PIP2 salt bridge plays critical roles in the *h*SK2 channel transition from the intermediate to the open conformational state.

The current study provides crucial insights into the atomistic structural and dynamic mechanisms of how PIP2 binding works in concert with Ca^2+^-CaM to activate the *h*SK2 channel. Indeed, the critical role of the R395 residue identified in this study is supported by its location close to the intracellular hydrophobic gate, conservation of the residue in all SK channels, and its dynamic formation of a salt bridge with residue E398 and PIP2 in the intermediate state of *h*SK2. Mutational studies of R395C-E398C tandem construct show that the mutant channel fails to be activated by PIP2 compared to the WT constructs, likely due to the neutralization of the R395 amino acid residue side chain and possibly the formation of intersubunit disulfide bonds in the double mutant channels, supporting the critical roles of the salt bridge disruption by PIP2 in the activation of *h*SK2 channels.

### Future Studies.

Our studies further raise important questions whether PIP2 binds residue R395 in closed or open states in addition to the intermediate state as observed in the current study. We observed that the flexibility of the hinge region at the C-terminal side of the S6 transmembrane segment near residue E398 is increased, when the salt bridge with the R395 residue is disrupted. However, this increase in flexibility needs to be further quantified with additional MD simulations. Further computational studies such as the string method with the swarm of trajectories (69) may also enable to observe the direct transition from intermediate to open state of the channel. In addition, one prior study of MD simulations of SK2 channel with its activator CyPPA demonstrated that CyPPA docked between CaM C lobe and the HA/HB helices of the channel widens the cytoplasmic gate of SK2 channels, with 2 activator molecules bound to the opposite subunits having the largest effect (70). Indeed, in our MD simulations, we observed asymmetric changes in the cytoplasmic gate upon binding of PIP2 to the amino acid residue R395 (*SI Appendix*, Fig. S7 and Table S2). In addition, there is an overall increase in the geometric average gate distances with PIP2 bound to the channels comparing to no PIP2 in the membrane. However, only one PIP2 molecule was bound to the R395 residue in our simulations since PIP2 was randomly distributed in the lower leaflet of the lipid bilayers, and binding was found to be a slow process even on a multimicrosecond-long scale of our MD simulations. Therefore, in future studies docking of 1 to 4 PIP2 molecules to the SK2 channel followed by MD simulations of these docked structures [as was done previously for a similar system (70)] will be performed to further test the widening of the cytoplasmic gate.

MD simulations of SK1-3 and SK4 channels will provide further insights into the distinct mechanisms of PIP2 activation of these channels. Finally, MD simulations using the current model on SK1-4 channels with activators or inhibitors will help to further our understanding of their atomistic-level mechanisms for the channel function modulation. Therefore, our study provides crucial mechanistic insights into PIP2 activation of the *h*SK2 channel and platforms for future structure-based drug designs for therapeutic inhibitors and activators of the SK channel family.

## Supplementary Material

Appendix 01 (PDF)

Movie S1. **K^+^ ion conduction through the open-state homology model of the hSK2 channel during MD simulations.**. A transmembrane voltage of 750 mV was applied with 150 mM of K^+^ ions in aqueous solution, 4 K^+^ ions were initially placed in the central channel pore and 3 more K^+^ ions in the selectivity filter. Only 2 opposite subunits of the pore domain with S5-S6 transmembrane segments of the *h*SK2 channel are represented in ribbon format for clarity. The selectivity filter of the *h*SK2 channel (SIGYGD from N- to C-termini) are represented in ball-and-stick format to visualize amino acid side chains. Water molecules have been removed for better visualization of K^+^ ions. K^+^ atoms are represented as large spheres in various colors.

Movie S2. **Schematic depiction of the proposed mechanisms of PIP2 binding and activation of the *h*SK2 channel.**. PIP2 is represented as a red circle. Schematics for S1-S6 transmembrane segments of *h*SK2 subunits I-IV, calmodulin (CaM) binding domains (CaMBD), and CaM are shown in green, orange, and light blue, respectively. The S5-S6 pore domain is represented as a dark blue circle in the center of *h*SK2-CaM complex. Salt bridges between amino acid residues R395:E398 from the adjacent subunits are represented as white bars. Coloring choices were made to match **Figure 7** in the main text.

Movie S3. **PIP2 disruption of the amino acid residues R395:E398 salt bridge formation during MD simulations.**. Only two adjacent subunits of the *h*SK2 channel are shown in ribbon representation for clarity. CaM is also omitted for clarity. The amino acid residues R395 and E398 from the adjacent subunits are shown in ball-and-stick representation in blue and red for R395 and E398, respectively. Two PIP2 molecules are visualized occupying “activation” sites. PIP2 is represented as spheres with hydrogen, carbon, oxygen, and phosphorus atoms shown in white, light blue, red, and orange, respectively. Spotlight during the simulation indicates the time point at which the salt bridge between amino acid residues R395:E398 is disrupted when the amino acid residue R395 forms a salt bridge with the phosphate group on PIP2.

## Data Availability

All final study data are included in the article and/or *SI Appendix* with key molecular modeling, molecular dynamics simulation and analysis data files and scripts available to download from Dryad digital repository at https://doi.org/10.5061/dryad.ksn02v7dj (71).
