# Supplementary material for: Atomistic mechanisms of the regulation of small-conductance Ca2+-activated K+ channel (SK2) by PIP2
Source: Proc Natl Acad Sci U S A. 2024 Sep 17;121(39):e2318900121. doi: 10.1073/pnas.2318900121 (PMC11441529; doi:10.1073/pnas.2318900121)
Supplement: Supplementary file 1 — Appendix 01 (PDF) [file pnas.2318900121.sapp.pdf]

ONLINE SUPPLEMENTAL INFORMATION (SI) APPENDIX

Atomistic mechanisms of the regulation of small-conductance Ca^{2+}-activated K^+ channel (SK2) by PIP2

Ryan L. Woltz[1,*], Yang Zheng[1,*], Woori Choi[1], Khoa Ngo[2], Pauline Trinh[1], Lu Ren[3], Phung N. Thai[1], Brandon J. Harris[2], Yanxiao Han[2], Kyle C. Rouen[2], Diego Lopez Mateos[2], Zhong Jian[4], Ye Chen-Izu[4], Eamonn J. Dickson[2], Ebenezer N. Yamoah[5], Vladimir Yarov-Yarovoy[2,6], Igor Vorobyov[2,4**], Xiao-Dong Zhang[1**], Nipavan Chiamvimonvat[1,4,7,8**]

dd

[1]Department of Internal Medicine, Division of Cardiovascular Medicine,
University of California, Davis, Davis, CA 95616
[2]Department of Physiology and Membrane Biology, UC Davis, Davis, CA, 95616
[3]Stanford Cardiovascular Institute, Stanford University, Stanford, CA 94305
[4]Department of Pharmacology, University of California, Davis, Davis, CA 95616
[5]Department of Translational Neuroscience,
University of Arizona College of Medicine, Phoenix, AZ 85004
[6]Department of Anesthesiology and Pain Medicine, University of California, Davis, Sacramento, CA 95817
[7]Department of Veterans Affairs, Northern California Health Care System, Mather, CA 95655
[8]Department of Basic Medical Sciences, University of Arizona College of Medicine,
Phoenix, AZ 85004

Running title: Mechanisms of SK2 channel regulation by PIP2

* Denotes co-first authors
** Denotes co-corresponding authors

Corresponding Authors:
Igor Vorobyov, Ph.D., Xiao-Dong Zhang, Ph.D., Nipavan Chiamvimonvat, M.D.
Department of Physiology and Membrane Biology,
And Department of Internal Medicine, Division of Cardiovascular Medicine
University of California, Davis
451 Health Science Drive, GBSF 6315
Davis, CA 95616
Department of Veterans Affairs, Northern California Health Care System
10535 Hospital Way Mather, CA 95655
E-mails: ivorobyov@ucdavis.edu; xdzhang@ucdavis.edu; nchiamvimonvat@ucdavis.edu

MATERIALS AND METHODS

Plasmid construction

Human filamin A (FLNA) in pREP4 vector (Life Technologies, Carlsbad, CA) was a kind gift from Dr. Paramita M. Ghosh (UC Davis, Davis, CA). α-Actinin2 cDNA in pcDNA3 vector was a kind gift from Dr. David Fedida (University of British Columbia, Canada) (1). Construction of *h*SK2 expression plasmids for heterologous expression in Chinese hamster ovary **(**CHO) was as follows: full-length human cardiac SK2 cDNA was subcloned into pIRES2-EGFP (Takara Bio USA, Inc., Mountain View, CA) to obtain pSK2-IRES-EGFP plasmid.

Optogenetic constructs

Optogenetic approaches were first tested in CHO cells transfected with *h*SK2, α-actinin2, and FLNA plasmids. The biomolecular probes were previously developed and described (2) (main-text **Fig. 1A**). The system is based upon dimerization between two proteins. The transcription factor CIBN is fused to CAAX, a plasma membrane anchor. *Arabidopsis thaliana* cryptochrome 2 (mCherry-CRY2) is fused to a PI(4,5)P2 5-phosphatase (5P) that is cytoplasmic at rest and is recruited to the CIBN-CAAX anchor upon the application of blue light (458 nm), thus bringing the phosphatase into close proximity with PIP2, resulting in dephosphorylation and subsequent depletion of PIP2 within minutes (main-text **Fig. 1A**).

To assess the validity of our results, control experiments were performed. Cells were transfected with either the CIBN or CRY2 constructs as a negative control or CRY2 fused to PI(4,5)P2 5P containing a point mutation in the catalytic domain rendering it inactive (5P-Dead) (main-text **Fig. 1**). Data were analyzed in a blinded fashion. We have successfully used the technique to induce clustering of K_V7.4 channels (3).

Chinese Hamster Ovarian (CHO) and Human Embryonic Kidney (HEK) 293 cells and plasmid transfection

CHO and HEK 293 cells were maintained in Ham's F-12 medium and Dulbecco's Modified Eagle medium (DMEM), respectively, supplemented with 10% fetal bovine serum and 1% penicillin-streptomycin in a humidified atmosphere containing 5% CO_2 at 37 °C. All cell culture reagents were purchased from Life Technologies. CHO cells were transfected using the following plasmid compositions: pSK2-IRES-EGFP, in combination with pREP4-FLNA and pcDNA3-α-actinin2, with optogenetic constructs, using Lipofectamine™ 2000 (Life Technologies) according to the manufacturer's protocol. The 1:1 ratio of the plasmids was determined to be most optimal for SK2 current expression in our prior published studies (4).

In order to test the prediction of the critical roles of R395:E398 salt bridge on PIP2 regulation of *h*SK2 channel, we generated a tandem construct of two *h*SK2 subunits containing R395C mutation in subunit 1 and E398C mutation in subunit 2 (generated and sequence verified by VectorBuilder). The wild-type (WT) construct was used as control. HEK293 cells were transiently transfected with the WT or the mutant tandem constructs in combination with pREP4-FLNA and pcDNA3-α-actinin2, using FUGENE (Promega) following the manufacturer's protocol. The cells were transfected for 48h and maintained in medium without antibiotics during transfection.

Isolation of ventricular myocytes from rabbit heart

All animal care and procedures were approved by UC Davis Institutional Animal Care and Use Committee and was in accordance with NIH and institutional guidelines. For isolation of the rabbit ventricular myocytes, New Zealand White rabbits (males, 3–4 months old, 2.5-3 kg) were first injected with heparin (5000 U) and then anesthetized with isoflurane (5%). After achieving

deep anesthesia, a standard enzymatic technique was used to isolate ventricular myocytes at 37 °C. Briefly, hearts were mounted on a Langendorff system and retrogradely perfused for 5 min with oxygenated solution containing (in mmol/l): NaCl 138, KCl 5.4 , $CaCl_2$ 0.05, $MgCl_2$ 1, NaH_2PO_4 0.33, 10 $NaHCO_3$, N-2-hydroxyethylpiperazine-N-2-ethane sulfonic acid (HEPES) 10, glucose 6, pyruvic acid 2.5, pH = 7.4. When blood was removed from the coronary circulation, the solution was supplemented with 1 mg/ml type II collagenase (305 U/mg; Worthington Biochemical Co., Lakewood, NJ, USA), 0.05 mg/ml protease type XIV (Sigma-Aldrich Co., St. Louis, MO, USA) and 1 mg/ml bovine serum albumin. The heart was perfused for 30 min to enzymatically dissociate cells. Portions of the left ventricular wall were cut into small pieces and the cell suspension was washed with the above solution. Finally, the Ca^{2+} concentration was gradually restored to 1.2 mM.

Transfection of rabbit ventricular myocytes using magnetic nanoparticles

Transfection of cardiomyocytes is much more complicated than that of cell lines and was achieved using magnetic nanoparticles as we have previously described (5). We have observed a transfection efficiency in cardiomyocytes of ~43 ± 2% in our recent study (5).

Patch-clamp recordings

Whole-cell $I_{K,Ca}$ was recorded from transfected CHO and HEK 293 cells at room temperature using conventional patch-clamp techniques, as previously described (6). The recordings were conducted with Axopatch 200B amplifier and Digidata 1320 digitizer (Molecular Devices). The protocols were generated with pCLAMP 10.3 software (Molecular Devices). The extracellular solution contained (in mmol/liter (mM)): *N*-methylglucamine (NMG) 140, KCl 4, $MgCl_2$ 1, glucose 5, and HEPES 10, pH 7.4 with HCl. The internal solution contained (in mM): potassium gluconate 144, $MgCl_2$ 1.15, EGTA 5, HEPES 10, and $CaCl_2$ yielding a free cytosol Ca^{2+} concentration of 500 nM, using the software by C. Patton of Stanford University

(http://maxchelator.stanford.edu/). The pH was adjusted to 7.25 using KOH. To isolate apamin-sensitive $I_{K,Ca}$, extracellular solution containing apamin (10 nM) was applied during the recordings, and the difference currents between the control and the apamin-containing solution were calculated. The current was elicited from a holding potential of -55 mV using a ramp voltage-clamp protocol ranging from -120 to +60 mV with a 2 second duration. diC_8-PI(4,5)P2 (10 nM, Echelon Biosciences) was applied to the intracellular patch-clamp solution to increase PIP2 concentrations.

For recording $I_{K,Ca}$ from rabbit ventricular myocytes, the extracellular solution contained (in mM): NMG 130, KCl 1, glucose 10, 4-amiopyridine 5, $CaCl_2$ 2, niflumic acid 0.05, E4031 0.001, chromanol 0.01, $BaCl_2$ 0.05, and HEPES 10, pH 7.4 with glutamic acid. The pipette solution consisted of (in mM): KCl 45, NMG 120, 10 HEPES, EGTA 0.05, pH 7.25 adjusted by glutamic acid. The voltage-clamp protocol was designed to isolate apamin-sensitive $I_{K,Ca}$ as shown in main-text **Fig. 1** activated by Ca^{2+} influx and Ca^{2+} released from the sarcoplasmic reticulum as we have previously described.

All recordings were performed using 3 M KCl agar bridges. Cell capacitance was calculated as the ratio of total charge (the integrated area under the current transient) to the magnitude of the pulse (20 mV). Currents were normalized to cell capacitance to obtain the current density. The series resistance was compensated electronically. In all experiments, a series resistance compensation of ≥90% was obtained. The currents and membrane potentials were recorded using Axopatch 200A amplifier (Molecular Devices, LLC., Sunnyvale, CA, USA), filtered at 1 kHz using a 4-pole Bessel filter and digitized at a sampling frequency of 5 kHz. Data acquisition and analysis were carried out using pClamp 10 software (Molecular Devices) and Origin Software (OriginLab, Northampton, MA, USA).

Statistical Analysis

Statistical significance for the experimental data was determined using unpaired two-tailed Student's t-test. $p<0.05$ was considered statistically significant.

Computational modeling of *h*SK2 channel to generate starting structures for MD simulations

Generation of structural models for *h*SK2 channel was achieved *via* three stages using *Rosetta* molecular modeling (**Online Supplemental Fig. S1**) as described below.

Electron density map refinement:

In the first stage, we refined and converted three states of the *h*SK4-CaM cryo-EM structures (PDB IDs: 6CNM, 6CNN, and 6CNO) into *Rosetta*-optimized energy with *Rosetta* 2021 software. Cryo-EM refinement was performed with side chains only being optimized. This conversion facilitated the energy terms of these structures to align with *Rosetta*'s scoring function (Score.gd2), leading to improved homology modeling. We used a modified version of the *Rosetta* demo included within the Rosetta software (https://new.rosettacommons.org/demos/latest/public/electron_density_structure_refinement/structure_refinement) (7, 8).

Homology modeling of hSK2 channels in closed, intermediate, and open states

In the second stage, we employed the modified *Rosetta* Comparative Modeling (*RosettaCM*) protocol to generate *h*SK2 homology models (8-15). This involved conducting sequence alignments between *h*SK4 and *h*SK2 channels, and subsequently formatting the aligned sequences into a Grishin format suitable for *RosettaCM*. Regions in *h*SK2 that were not present in *h*SK4 structures were modeled using the loop modeling protocol, (https://www.rosettacommons.org/docs/latest/application_documentation/structure_prediction/lo

op_modeling/KIC_with_fragments) (16) primarily the S3-S4 linker. The template protocol can be found at this link: (https://new.rosettacommons.org/demos/latest/Home).

The top model from the cryo-EM refinement of *h*SK4 step was then used as the template for homology modeling for *h*SK2. The first attempt was to only model *h*SK2, then dock CaM onto the channel. However, the absence of CaM in the models triggered large movements in the CaM binding domain (CaMBD) in the C-terminal domain of *h*SK2 (main-text **Fig. 2A**) due two factors: A) CaMBD is perpendicular to the S1-S6 transmembrane segments of *h*SK2 and B) the linker region that connects the CaMBD and S1-S6 is very flexible. In contrast, the inclusion of CaM led to convergence and improved agreement in the top models for the CaMBD (main-text **Fig. 2B-D**), resulting in a model that is much closer to the template.

In our attempt to create reliable homology models, several features were included in the protocol, namely implicit lipid membrane environment to accurately model membrane-spanning protein segments, enforcing symmetry to preserve a four-fold homotetrameric *h*SK2-CaM complex symmetry, explicit inclusion of metal ions to preserve the Ca^{2+} binding loops, treatment of multiple chains that include *h*SK2 and CaM, and loop modeling for the flexible regions of the protein missing from the cryo-EM structures. However, an error occurred between the symmetry function and the metal binding feature, specifically, the Ca^{2+} ions assumed the exact coordinates of the first C_α atom of the first residue of CaM. This necessitated the homology modeling to be performed without the symmetry function.

In addition, we meticulously monitored for possible displacement of the backbone carbonyl oxygen atoms in the selectivity filter (SF) of *h*SK2 during the homology modeling since deformation of the SF may lead to a non-conducting channel in the MD simulations. Indeed, repulsion of the oxygen atoms results in the SF deformation at amino acid residues I359 with

widening and shortening of the SF. This necessitated the inclusion of harmonic restraints that were determined empirically, and a weighted value of 100.0 kcal/mol/Å^2 was used on the C_α atoms of the backbone of each amino acid residue in the SF. Additionally, the deformation was minimized with explicit inclusion of K^+ ions in the SF. Interestingly, we did observe that the open state of *h*SK2 required the largest restraints to maintain the SF structure. 50,000 models were created for each conformational state of *h*SK2-CaM, and a top model was selected with a standard clustering selection process.

Clustering and top model selection:

Standard *Rosetta* clustering was performed for each cryo-EM refinement and homology modeling step with minor differences in the filtering process, based on the models that were used as inputs. The cryo-EM refinement output structures were filtered first by sorting using the "*r15*" term (REF2015 terms: *SCORE – elec_dens_fast*) and keeping the lowest 50% of *r15* ranked decoys. The resulting list of decoys was then sorted *by elec_dens_fast* term, and the lowest 20% were kept. This final selection was then clustered with a radius determined empirically to provide a distribution, where the most decoys (~30%) were in the first cluster, and subsequent clusters were with progressively fewer decoys. An attempt to obtain upwards of 90% of all models in the first 20 clusters was made. The centers of the top 10 clusters were then evaluated for retention of similar critical structural features described above. The top model from these 10 was used as a template for the homology modeling. The results of the homology modeling were sorted by the total *score* term with the lowest 10% used for clustering. Clustering of the homology models was performed in the same manner as the cryo-EM refinement with the top model used as the starting structure for MD simulations.

Molecular dynamics (MD) simulations

The final stage involved molecular dynamics (MD) simulations. The *h*SK2 models derived from homology modeling were initially visualized without a membrane and then embedded into 1-palmitoyl-2-oleoyl-sn-glycero-3-phosphocholine (POPC) lipid bilayer and solvated by 0.15 M KCl aqueous solution using CHARMM-GUI (17-19) (**Fig. 3A**). **Online Supplemental Table S1** provides a summary of 18 5-µs-long MD simulations on Anton 2 supercomputer (20) of *h*SK2-CaM complex in POPC membrane with or without mono-protonated state of phosphatidylinositol -(4,5)-bisphosphate (PIP2) with protonation on P4 oxygen atom (SAPI24) at 2.5, 5, and 10% in the lower leaflet of the lipid bilayer. The concentrations of PIP2 were chosen based on recent estimations of PIP2 in the lower leaflet of the lipid bilayer that can be as high as 2-5% (21). In addition, we performed a total of 27 simulations at 1 µs using either NAMD 3.0 alpha (22) or AMBER18 (23) on high performance computing (HPC) EXPANSE platform (San Diego Supercomputer Center at the University of California, San Diego) with computational time granted through Extreme Science and Engineering Discovery Environment, XSEDE (now Advanced Cyberinfrastructure Coordination Ecosystem: Services & Support, ACCESS).

MD simulations were run in the *NPT* ensemble at 310 K and 1 atm pressure using tetragonal periodic boundary conditions using a standard set of non-bonded cutoffs and other options as in our previous studies (24, 25). All-atom biomolecular CHARMM36m protein (26), C36 lipid (27, 28), and TIP3P water (29) were used. Each MD simulation system was equilibrated for 2.27 ns with suggested gradually diminishing positional and dihedral restraints provided by CHARMM-GUI scripts. Due to the relatively large size of the *h*SK2-CaM protein complex in our MD simulations and to ensure its conformational stability (as described above), a follow-up extended equilibration MD simulation was performed for 100 ns with gradually reduced restraints

on protein backbone atoms of the *h*SK2-CaM complex and its components as shown in main-text **Fig. 3B**. The extended equilibration protocol was performed on the EXPANSE platform with AMBER18 (**Fig. 3B**). The protein backbone restraints were maintained using the force constant of 1.0 kcal·mol^{-1}·Å^{-2} during the initial environment equilibration until the start of the extended equilibration stage, when the restraints were successively reduced in a 5 or 10 ns stepwise fashion, starting from the periphery of the protein and moving towards the center of the protein over a period of 100 ns (main-text **Fig. 3B**). The extended equilibration protocol was determined empirically to maintain the stability of essential structural features such as the pore domain or SF. Each successive step of either 5 or 10 ns was determined based on root-mean-square deviation (RMSD) profiles reaching a plateau indicating system equilibration. Post 100 ns of this extended equilibration, the production MD simulation runs were performed for 1000 ns using AMBER18 on EXPANSE or for 5000 ns on Anton 2. Small restraints were used on the C_α backbone atoms of the selectivity filter residues SIGYGD throughout the production stage (**Fig. 3B**)

The 27 MD simulations created were divided as follows: 3 distinct *h*SK2 conformational states (closed, intermediate, and open) embedded into POPC:PIP2 complex membranes with PIP2 residing in the lower leaflet only (100:0, 95:5, 90:10). All simulations were run in triplicate to ensure consistency. The 18 most stable simulations of the 27 runs using AMBER18 were identified and transferred to Anton 2 by using molecular coordinates and velocities from the final frame of 100 ns protein equilibration stage as a starting point. The MD simulations on Anton 2 are unbiased 5-µs-long runs and are described in **Online Supplemental Table S1**.

The MD simulation trajectories were analyzed via root-mean-square deviation (RMSD) calculations (main-text **Fig. 3C**). The distinct initial channel states were characterized by central channel pore radius mapping using HOLE analysis script (30) (main-text **Fig. 4A-C**). We thus

monitored channel conformational states by measuring the minimum hydrophobic gate diameter for each frame (main-text **Fig. 4D-E**). This was performed by measuring the distance between side-chain $C_{\gamma 1}$ (CG1) atoms of the V390 residues on the pore-lining S6 helices from opposing subunits.

Ion conduction was measured by monitoring the *Z*-axis coordinates of K^+ ions through the SF (main-text **Fig. 4F-G**). We then quantified the number of ions that completed a connected path through the SF to avoid counting ions that would "jump" from the intracellular aqueous compartment to the extracellular one using periodic boundary conditions (PBC) image recentering. PIP2 movement was determined by tracking its *XY* coordinates and plotting 50 points per graph for each PIP2 molecule. The identification of all possible unbiased PIP2 binding sites was achieved using modified scripts from MD analysis GitHub (https://github.com/MDAnalysis/mdanalysis), (31) and amino acid residues of interest were located by identifying salt bridges formation between *h*SK2-CaM complex and PIP2 head group. Visual, structural, and computational analysis was completed using Visual Molecular Dynamics (VMD) (32), UCSF Chimera (33) and ChimeraX (34) (University of California San Francisco).

Clustering Analysis

After the completion of the MD simulations, we conducted a clustering analysis on the simulation data. The primary objective of this analysis was to categorize conformations based on their similarity and to pinpoint the most frequently occurring conformational states of *h*SK2. Additionally, this analysis aimed to investigate the coordination of *h*SK2 and PIP2 clusters and the formation of the R395:PIP2 salt bridge. To achieve these goals, we employed the TTClust program, a specialized tool specifically designed for trajectory clustering (35) (https://github.com/tubiana/TTClust).

We first aligned the PIP2 molecule with respect to the *h*SK2-CaM. The trajectory of the aligned PIP2 molecule was saved as a binary DCD trajectory file. Subsequently, we executed the TTClust program using the command: ttclust -f HETA-c.dcd -t HETA-c.pdb -sa "none" -sr "all". The -sa parameter was set to "none" to indicate no further alignment was required as it was already performed in the first step. The -sr parameter was set to "all" to select all atoms in the analysis. We then analyzed the clustering results to identify clusters with PIP2:R395 salt bridges, focusing on the major residues involved. We visualized and compared these clusters against the *h*SK2-CaM structure to observe their distribution and identify their location. Furthermore, we calculated the time point at which a salt bridge was detected via a 3.6 Å cutoff.

Solvent-Accessible Surface Area (SASA) analyses

Solvent-accessible surface area (SASA) analyses were performed to quantify the solvent exposure of each amino acid residue in the transient, transfer, and activation PIP2 binding sites from the closed, intermediate, and open states. There were no significant differences in the SASA over time or averaged SASA for amino acid residues within the same state for MD simulations with the *h*SK2-CaM embedded in a POPC or a POPC/PIP2 membrane if a PIP2 molecule did not directly interact with that amino acid residue. Therefore, we calculated the SASA for each amino acid residue on the four subunits for each frame based on binary classification: bound vs. unbound by PIP2 (the distance between any anionic oxygen on PIP2 head group and any cationic nitrogen on the side chain of basic amino acids was <4 Å was defined as bound and >4 Å was defined as unbound).

For the open *h*SK2 state, we further classified the MD simulations into 2 additional groups with an intact hydrophobic gate and a collapsed hydrophobic gate, which became nonconductive

after the initial K^+ ions in the channel pore were depleted. These simulations were excluded from the analyses since there were major asymmetry of the pore.

Online Supplemental Figures

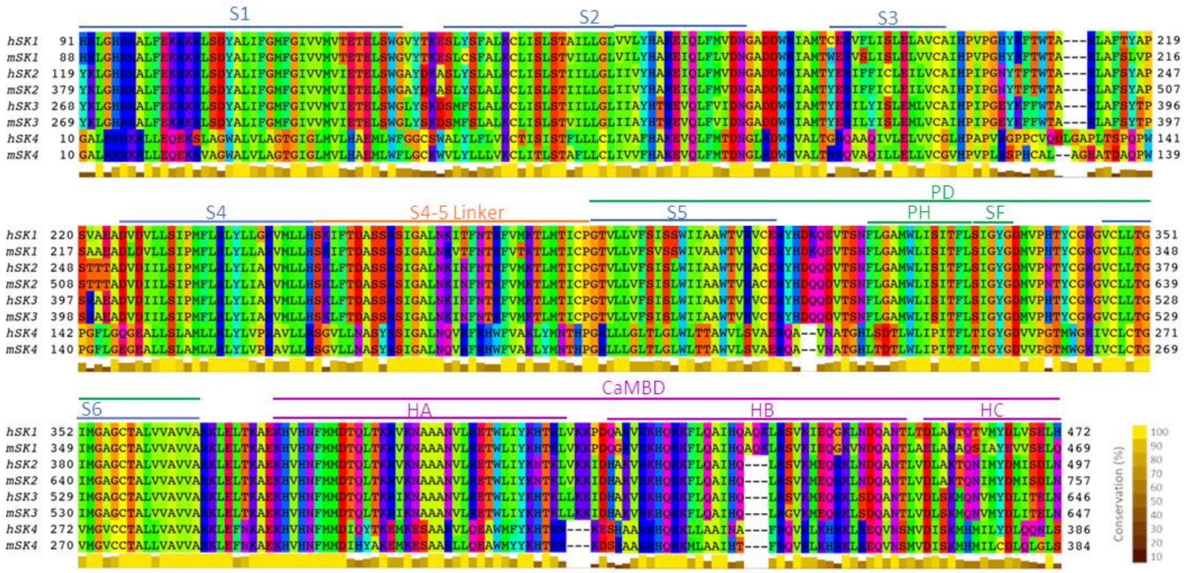

Online Supplemental Figure S1: Amino acid sequence alignment for human (*h*) and mouse (*m*) SK1-3 channels with percentages of conservation. S1-S6, SF, PD, S4-5 linker, CaMBD, HA, HB, and HC refer to transmembrane segments S1-S6, selectivity filter, pore domain, calmodulin binding domain, and helices A-C, respectively.

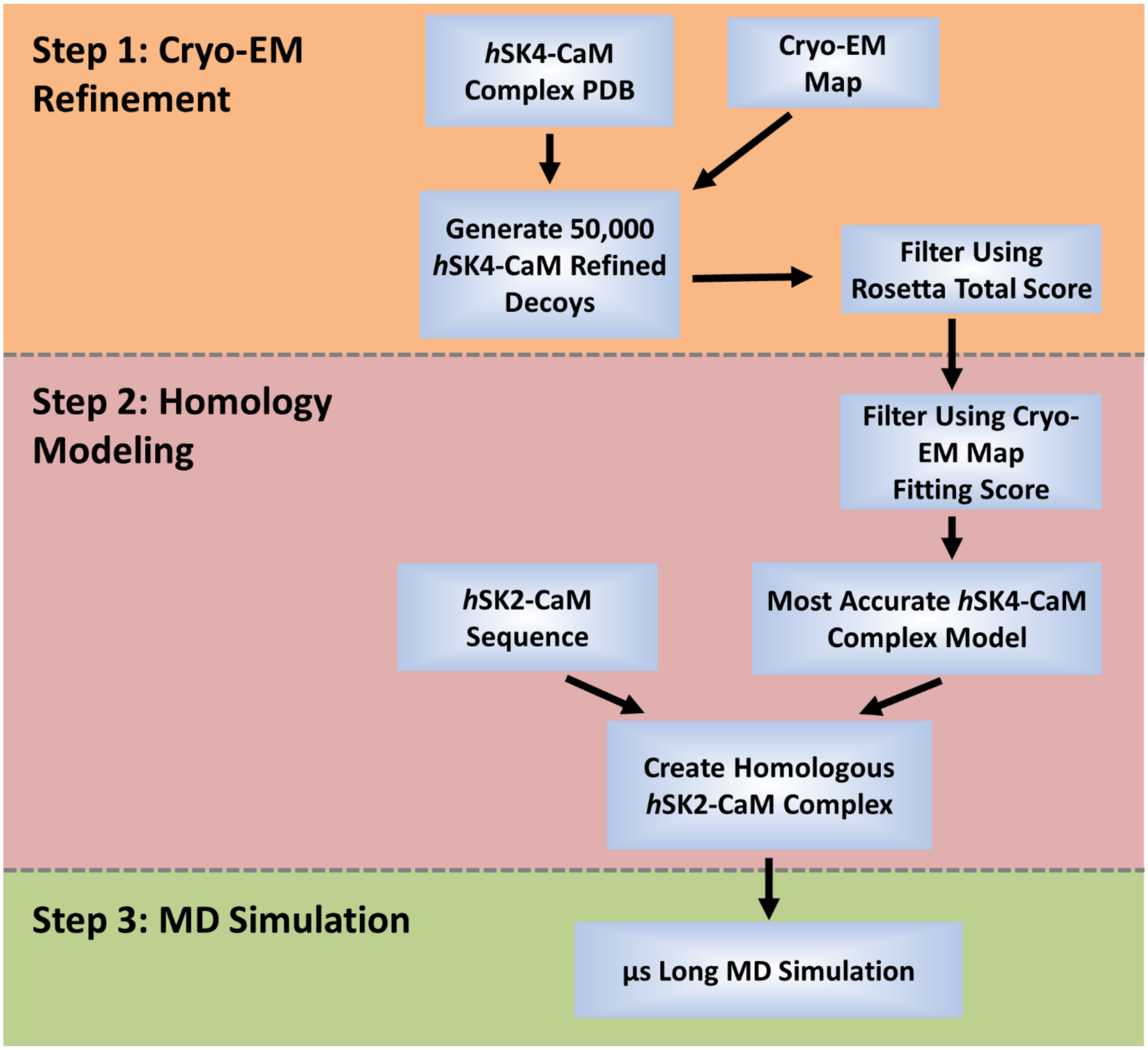

Online Supplemental Figure S2: Flowchart summarizing methods for the generation and simulation of *h*SK2 channel models for each conformational state. Step 1: Refinement and conversion of *h*SK4-CaM cryo-EM structures into *Rosetta* energy optimized structural models. **Step 2:** Modified *RosettaCM* protocol to generate *h*SK2-CaM homology model from *h*SK4-CaM refined cryo-EM structures. **Step 3:** MD equilibration and production protocol. All steps are described in detail in *Materials and Methods* section above.

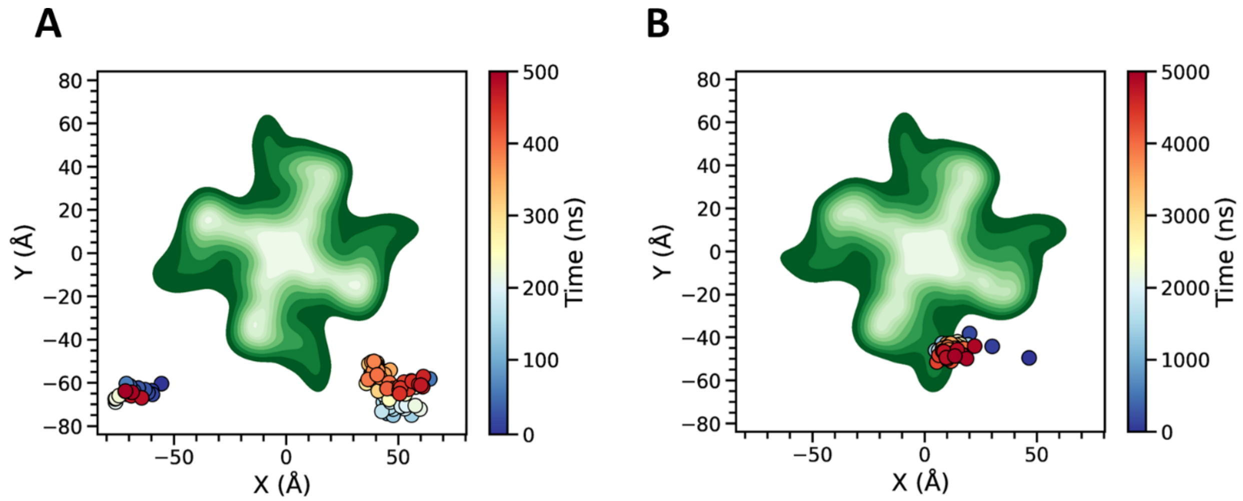

Online Supplemental Figure S3: Tracking of positions of PIP2 molecules over MD simulation time from the peripheral (bulk lipid membrane) to the *h*SK2 PIP2 binding sites. The *h*SK2-CaM tetramer is viewed from the extracellular side at the top.

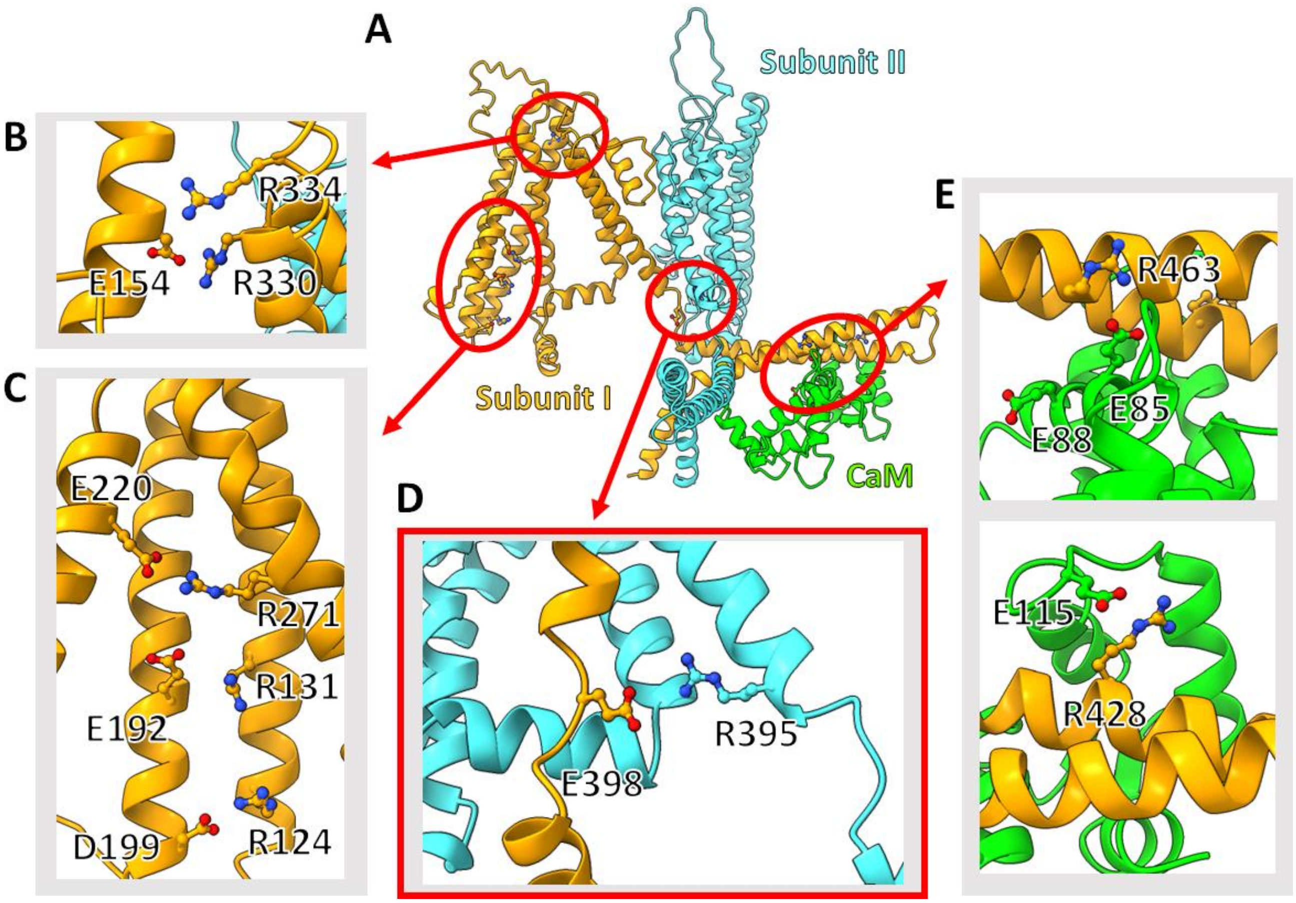

Online Supplemental Figure S4: Formation of salt bridges between different amino acid residues in the *h*SK2-CaM tetramer. A. Representative poses from clustering of MD simulations of *h*SK2-CaM channel, with only two subunits shown for clarity in ribbon format in orange and cyan colors. CaM molecule is shown in green. Insets in **B-D** are zoomed-in regions from panel **A** with side chains of key amino acid residues shown in ball-and-stick format with oxygen (red), nitrogen (blue), and carbon (orange, cyan, or green). Hydrogen atoms are not shown. **B-C.** Intra-subunit salt-bridge formation in the S1-S4 domain of in subunit I. C. **D.** Inter-subunit salt bridge formation between residue R395 from S6 transmembrane helix from subunit I and residue E398 from S6 transmembrane segment from the adjacent subunit II. **E.** Intra-subunit salt-bridge formation as well as salt-bridge formations with CaM.

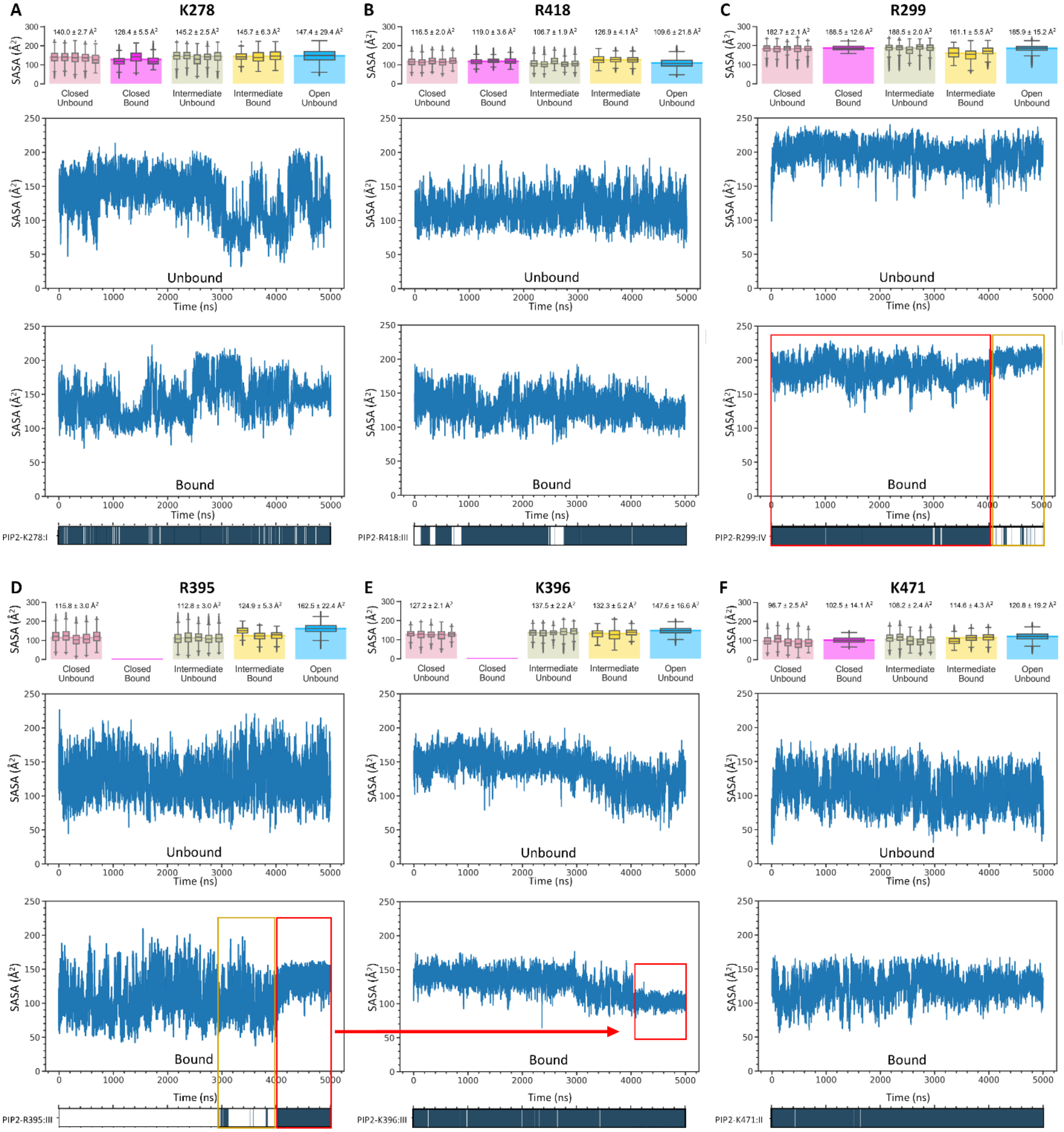

Online Supplemental Figure S5. Evaluations of simultaneous Solvent Accessible Surface Area (SASA) and salt bridge formations. **A-F**. SASA values with corresponding salt bridge formation during different 5-μs long unbiased MD simulations for amino acid residues K278, R418, R299, R395, K396, and K471, respectively, for PIP2 bound vs. unbound MD simulation frames of closed, intermediate, and open conformational states of *h*SK2-CaM. If the minimum distance between anionic oxygen on any PIP2 head group and the positively charged nitrogen on the side chains of basic amino acid residues was < 4 Å, it was defined as bound and > 4 Å was defined as unbound. The top panels in **A-F** show summary data presented as box-and-whisker

plots with the minimum value, the first quartile, the median, the third quartile, and the maximum value. The height of each box corresponds to standard deviation. The averages of all frames from all simulations in the same conformational state of the *h*SK2-CaM are presented as the shaded area. The two middle panels in panels **A-F** show the time series of SASA values (in Å^2) for the 5-μs-long MD simulations for the corresponding amino acid residues for the closed (**A**) and intermediate (**B-F**) states of the *h*SK2-CaM in the PIP2 unbound and bound states, respectively. The bottom panels in **A-F** show the time series of salt bridge formation between PIP2 and the corresponding amino acid residue.

Results: A-D. There are changes in the SASA values between the PIP2 unbound or bound states for amino acid residues K278 (**A**), R418 (**B**), R299 (**C**), R395 (**D**) with the largest changes for the residue K278 (**A**) in the closed state and the residues R299, R395, and R418 in the intermediate state **E-F**. Amino acid residues K396 and K471 exhibit very little change in SASA values upon binding by PIP2. Yellow box in panel **C** designates the simulation time when salt bridge is formed between amino acid residue R395 and PIP2, leading to a significant decrease in the standard deviation of the SASA value for residue R299. **D.** Quantification of the simultaneous SASA and the salt bridge formation for the residue R395 in the intermediate state of the *h*SK2-CaM. For the transient salt bridge formation between amino acid residue R395 and PIP2 (at ~3,000 ns of the MD simulation, marked by a yellow box), there is no notable feature in the time plot for SASA corresponding to the salt bridge formation despite an obvious rotamer change of this amino acid residue in the MD clustering results (main text, **Fig. 6**). In contrast, there is a simultaneous increase in the SASA with a significant decrease in the variance, occurring with a steady salt bridge formation (at ~4,000 ns of the MD simulation, marked by a red box). **E.** There are simultaneous changes in the SASA values of the adjacent K396 residue when residue R395 forms multiple salt bridges with PIP2 at ~4,000 ns of the MD simulation with a significant decrease in standard deviation, possibly due to concealment of the SASA of residue K396 by the R395 rotamers. The SASA for residue R395 in the intermediate state significantly increases upon binding by PIP2. Consequently, the SASA for the R395 residue exhibits a very large increase in the open state compared to the intermediate state. The salt bridge disruption between residues R395 and E398 residues in the open state further supports the notion that that the R395:PIP2 salt bridge plays critical roles in the *h*SK2 channel transition from an intermediate to an open state.

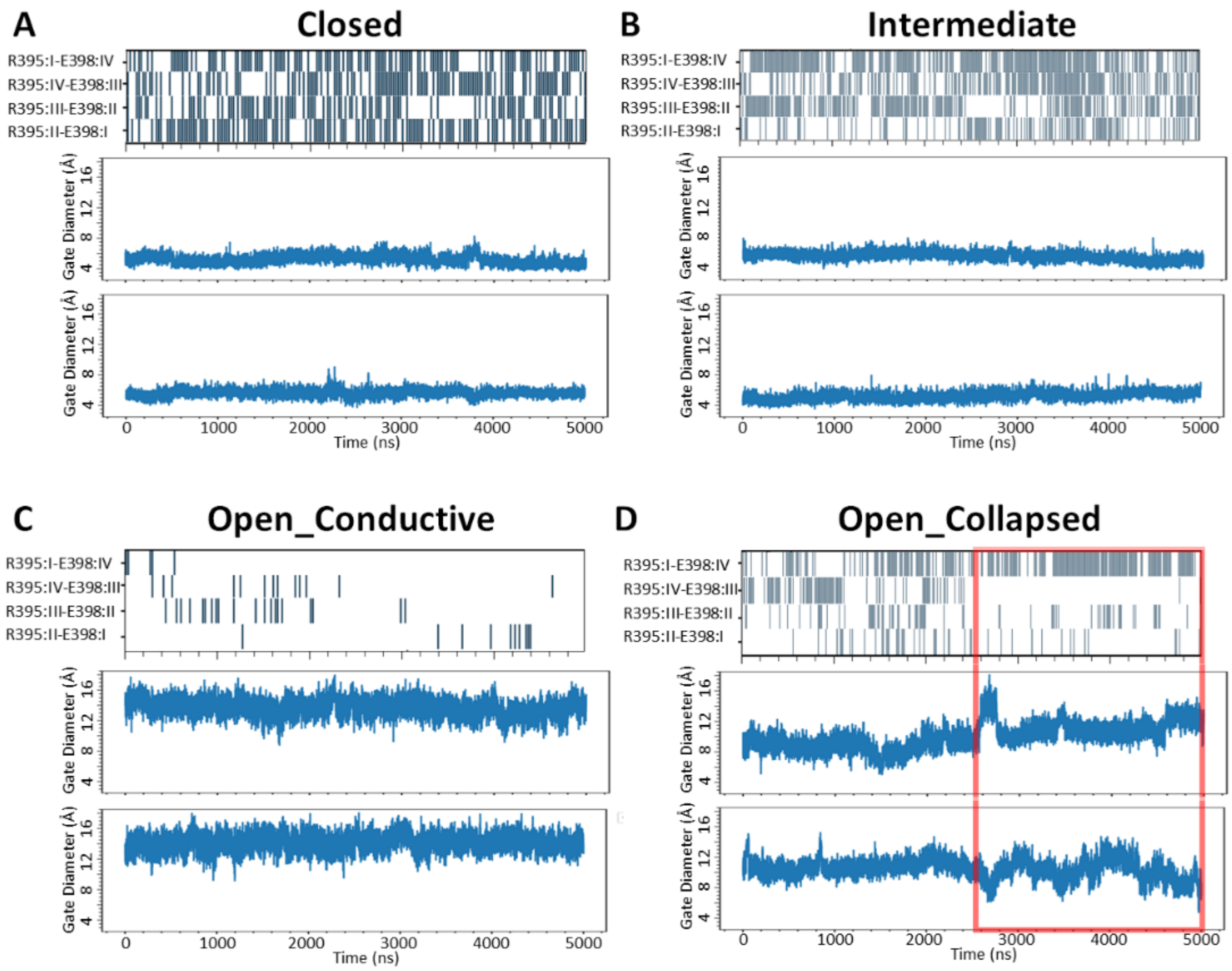

Online Supplemental Figure S6. There is a decrease in R395:E398 cross subunit salt bridge formation as the intracellular gate diameter increases. A-D. Time series of the salt bridge formation (top) between the amino acid residue R395 of the indicated subunit (I-IV) and the amino acid residue E398 of the adjacent subunit in different states: closed (**A**), intermediate (**B**), open and conductive (**C**), and open with the hydrophobic gate asymmetrically collapsed during the simulations (**D**, red box). The corresponding intracellular gate distances over simulation time were computed between the amino acid residue V390 C_α atoms on opposing subunits. Distance was measured between subunits I:III (middle panel) and II:IV (bottom panel). The collapse of the intracellular gate (**D**) shows an increase in the frequency of the R395:E398 salt bridge formation.

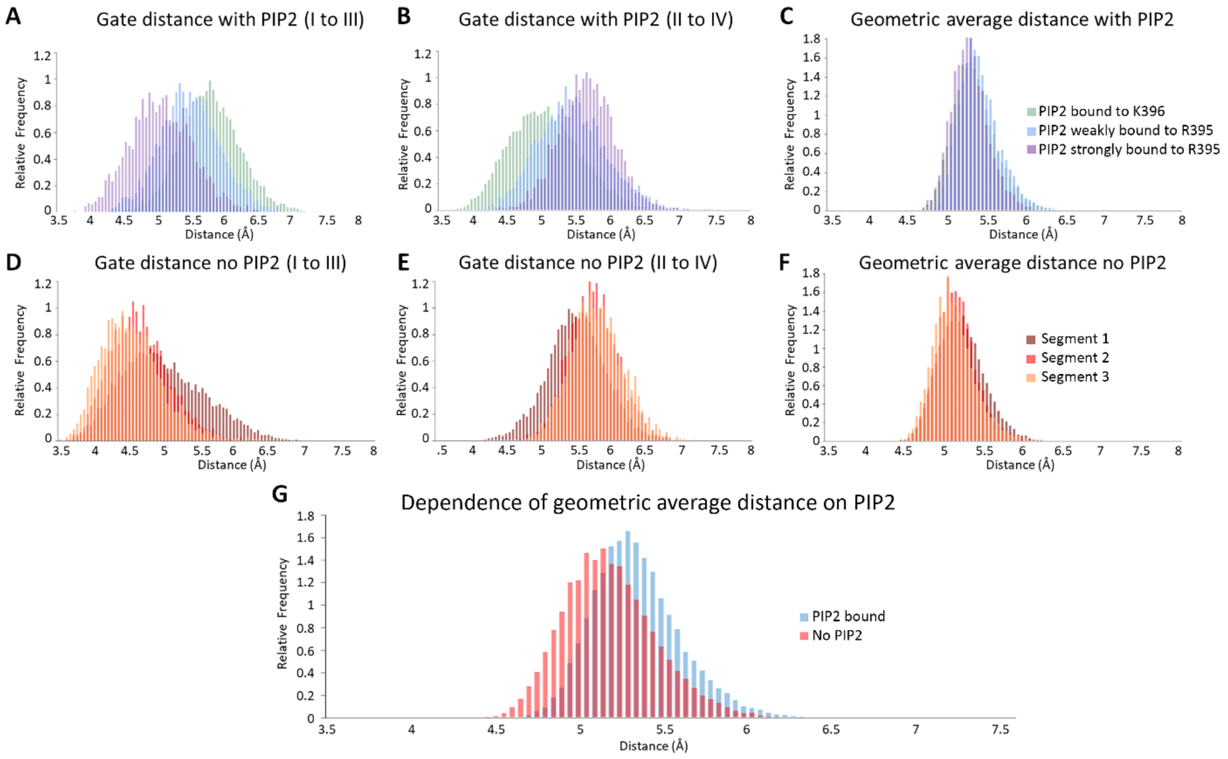

Figure S7: Effects of PIP2 binding on the cross-subunit diameter of the cytoplasmic hydrophobic gate at the V390 residues. The diameter of the cytoplasmic gate distance was determined between CG (C_{γ}) side-chain atoms of amino acid residues V390 of opposite subunits: I-III and II-IV. Histograms are plotted using 3.5-8.5 Å range with a bin size of 0.05 Å. **A-B.** Diameters were measured from the atoms in subunit I to III (**A**) and II to IV (**B**) in an MD simulation with 2 PIP2 molecules bound to the *"activation"* binding site. The simulation was divided into 3 segments based on the clustering of PIP2 binding to the amino acid residue R395 identified in ***Figure 6 (main text)***: 1) PIP2 bound to the *"activation"* binding site (amino acid residue K396 but not the amino acid residue R395, 1-2,950 ns) is shown in green; 2) One PIP2 weakly bound to the amino acid residue R395 (2,950-4,050 ns) is shown in blue; 3) The same PIP2 strongly bound to the amino acid residue R395 (with multiple salt bridge formed, 4,050-5,000 ns) is shown in purple. **C.** The geometric average is calculated by taking the square root of the product of the diameters for subunits I to III and II to IV for every MD simulation frame to assess the asymmetry of the gate distances. **D-F**. Same analyses as in **A-C** for an MD simulation containing no PIP2 were performed as controls. The simulation was also divided into three segments: segment 1 (1-2,950 ns) shown in brown, segment 2 (2,950-4,050 ns) shown in red, and segment 3 (4,050-5,000 ns) shown in orange. **G**. Geometric average diameters for MD simulations with bound PIP2 (**A-C**) compared to no PIP2 (**D-F**). Mean ± standard deviation (STD) values are shown in **Online Supplemental Table 2**.

Online Supplemental Table S1. A series of 18 MD simulations of the *h*SK2-CaM systems executed using Anton 2.

Channel state/replication number	Membrane environment/applied voltage	Size (atoms)	Simulation time (μs)	Anton days
Open/1	POPC	~290,000	5	0.5
Open/1	POPC:SAPI24 (95:5)	~290,000	5	0.5
Open/1	POPC:SAPI24 (97.5:2.5)	~290,000	5	0.5
Open/1	POPC/750 mV	~290,000	5	0.5
Open/1	POPC:SAPI24 (97.5:2.5)/750 mV	~290,000	5	0.5
Open/2	POPC	~290,000	5	0.5
Open/2	POPC:SAPI24 (95:5)	~290,000	5	0.5
Open/2	POPC/750 mV	~290,000	5	0.5
Intermediate/1	POPC	~290,000	5	0.5
Intermediate/1	POPC:SAPI24 (95:5)	~290,000	0.5	0.05
Intermediate/1	POPC:SAPI24 (95:5)	~290,000	5	0.5
Intermediate/2	POPC	~290,000	5	0.5
Intermediate/2	POPC:SAPI24 (95:5)	~290,000	5	0.5
Closed/1	POPC	~287,000	5	0.5
Closed/1	POPC:SAPI24 (95:5)	~287,000	5	0.5
Closed/1	POPC:SAPI24 (90:10)	~287,000	0.5	0.05
Closed/2	POPC	~287,000	5	0.5
Closed/2	POPC:SAPI24 (95:5)	~287,000	5	0.5

Online Supplemental Table S2. Effects of PIP2 binding on the cross-subunit diameter of the cytoplasmic hydrophobic gate at the V390 residues: Mean ± Standard Deviation (STD) from data shown in Fig. S7.

Gate and Geometric Average Distances	Mean ± STD (Å)
A) Gate distance with PIP2 (I to III)	
PIP2 bound to K396	5.78 ± 0.46
PIP2 weakly bound to R395	5.48 ± 0.45
PIP2 strongly bound to R395	5.05 ± 0.48
B) Gate distance with PIP2 (II to IV)	
PIP2 bound to K396	5.04 ± 0.52
PIP2 weakly bound to R395	5.42 ± 0.51
PIP2 strongly bound to R395	5.63 ± 0.43
C) Geometric average distance with PIP2	
PIP2 bound to K396	5.38 ± 0.28
PIP2 weakly bound to R395	5.43 ± 0.26
PIP2 strongly bound to R395	5.32 ± 0.24
D) Gate distance no PIP2 (I to III)	
No PIP2 segment 1	5.02 ± 0.61
No PIP2 segment 2	4.67 ± 0.42
No PIP2 segment 3	4.57 ± 0.47
E) Gate distance no PIP2 (II to IV)	
No PIP2 segment 1	5.53 ± 0.43
No PIP2 segment 2	5.78 ± 0.36
No PIP2 segment 3	5.83 ± 0.39
F) Geometric average distance no PIP2	
No PIP2 segment 1	5.25 ± 0.30
No PIP2 segment 2	5.19 ± 0.25
No PIP2 segment 3	5.15 ± 0.27
G) Geometric average distance dependence on PIP2	
No PIP2	5.22 ± 0.29
With PIP2	5.38 ± 0.27

Online Supplemental Movies:

Online Supplemental Movie M1: K^+ ion conduction through the open-state homology model of the *h*SK2 channel during MD simulations. A transmembrane voltage of 750 mV was applied with 150 mM of K^+ ions in aqueous solution, 4 K^+ ions were initially placed in the central channel pore and 3 more K^+ ions in the selectivity filter. Only 2 opposite subunits of the pore domain with S5-S6 transmembrane segments of the *h*SK2 channel are represented in ribbon format for clarity. The selectivity filter of the *h*SK2 channel (SIGYGD from N- to C-termini) are represented in ball-and-stick format to visualize amino acid side chains. Water molecules have been removed for better visualization of K^+ ions. K^+ atoms are represented as large spheres in various colors.

Online Supplemental Movie M2: Schematic depiction of the proposed mechanisms of PIP2 binding and activation of the *h*SK2 channel. PIP2 is represented as a red circle. Schematics for S1-S6 transmembrane segments of *h*SK2 subunits I-IV, calmodulin (CaM) binding domains (CaMBD), and CaM are shown in green, orange, and light blue, respectively. The S5-S6 pore domain is represented as a dark blue circle in the center of *h*SK2-CaM complex. Salt bridges between amino acid residues R395:E398 from the adjacent subunits are represented as white bars. Coloring choices were made to match **Figure 7** in the main text.

Online Supplemental Movie M3: PIP2 disruption of the amino acid residues R395:E398 salt bridge formation during MD simulations. Only two adjacent subunits of the *h*SK2 channel are shown in ribbon representation for clarity. CaM is also omitted for clarity. The amino acid residues R395 and E398 from the adjacent subunits are shown in ball-and-stick representation in blue and red for R395 and E398, respectively. Two PIP2 molecules are visualized occupying *"activation"* sites. PIP2 is represented as spheres with hydrogen, carbon, oxygen, and phosphorus atoms shown in white, light blue, red, and orange, respectively. Spotlight during the simulation indicates the time point at which the salt bridge between amino acid residues R395:E398 is disrupted when the amino acid residue R395 forms a salt bridge with the phosphate group on PIP2.

REFERENCES

1. D. Cukovic, G. W. Lu, B. Wible, D. F. Steele, D. Fedida, A discrete amino terminal domain of Kv1.5 and Kv1.4 potassium channels interacts with the spectrin repeats of alpha-actinin-2. *FEBS Lett* **498**, 87-92 (2001).
2. O. Idevall-Hagren, E. J. Dickson, B. Hille, D. K. Toomre, P. De Camilli, Optogenetic control of phosphoinositide metabolism. *Proc Natl Acad Sci U S A* **109**, E2316-2323 (2012).
3. M. C. Perez-Flores *et al.*, Cooperativity of Kv7.4 channels confers ultrafast electromechanical sensitivity and emergent properties in cochlear outer hair cells. *Sci Adv* **6**, eaba1104 (2020).
4. S. Rafizadeh *et al.*, Functional interaction with filamin A and intracellular Ca^{2+} enhance the surface membrane expression of a small-conductance Ca^{2+}-activated K^{+} (SK2) channel. *Proc Natl Acad Sci U S A* **111**, 9989-9994 (2014).
5. M. A. Yamoah *et al.*, Highly efficient transfection of human induced pluripotent stem cells using magnetic nanoparticles. *Int J Nanomedicine* **13**, 6073-6078 (2018).
6. Y. Xu *et al.*, Molecular identification and functional roles of a Ca^{2+}-activated K^{+} channel in human and mouse hearts. *The Journal of biological chemistry* **278**, 49085-49094 (2003).
7. H. Park *et al.*, Simultaneous Optimization of Biomolecular Energy Functions on Features from Small Molecules and Macromolecules. *J Chem Theory Comput* **12**, 6201-6212 (2016).
8. R. F. Alford *et al.*, The Rosetta All-Atom Energy Function for Macromolecular Modeling and Design. *J Chem Theory Comput* **13**, 3031-3048 (2017).
9. V. Yarov-Yarovoy, D. Baker, W. A. Catterall, Voltage sensor conformations in the open and closed states in ROSETTA structural models of K(+) channels. *Proc Natl Acad Sci U S A* **103**, 7292-7297 (2006).
10. V. Yarov-Yarovoy, J. Schonbrun, D. Baker, Multipass membrane protein structure prediction using Rosetta. *Proteins* **62**, 1010-1025 (2006).
11. C. A. Rohl, C. E. Strauss, K. M. Misura, D. Baker, Protein structure prediction using Rosetta. *Methods Enzymol* **383**, 66-93 (2004).
12. J. Subbotina *et al.*, Structural refinement of the hERG1 pore and voltage-sensing domains with ROSETTA-membrane and molecular dynamics simulations. *Proteins* **78**, 2922-2934 (2010).
13. V. Yarov-Yarovoy *et al.*, Structural basis for gating charge movement in the voltage sensor of a sodium channel. *Proc Natl Acad Sci U S A* **109**, E93-102 (2012).
14. R. Y. Wang *et al.*, Automated structure refinement of macromolecular assemblies from cryo-EM maps using Rosetta. *Elife* **5** (2016).
15. B. J. Bender *et al.*, Protocols for Molecular Modeling with Rosetta3 and RosettaScripts. *Biochemistry* **55**, 4748-4763 (2016).
16. D. J. Mandell, E. A. Coutsias, T. Kortemme, Sub-angstrom accuracy in protein loop reconstruction by robotics-inspired conformational sampling. *Nat Methods* **6**, 551-552 (2009).
17. S. Jo, T. Kim, V. G. Iyer, W. Im, CHARMM-GUI: a web-based graphical user interface for CHARMM. *J Comput Chem* **29**, 1859-1865 (2008).

18. E. L. Wu *et al.*, CHARMM-GUI Membrane Builder toward realistic biological membrane simulations. *J Comput Chem* **35**, 1997-2004 (2014).
19. J. Lee *et al.*, CHARMM-GUI Input Generator for NAMD, GROMACS, AMBER, OpenMM, and CHARMM/OpenMM Simulations Using the CHARMM36 Additive Force Field. *J Chem Theory Comput* **12**, 405-413 (2016).
20. D. E. e. a. Shaw (2014) Anton 2: Raising the Bar for Performance and Programmability in a Special-Purpose Molecular Dynamics Supercomputer. in *SC '14: Proceedings of the International Conference for High Performance Computing, Networking, Storage and Analysis, New Orleans, LA, USA* (New Orleans, LA, USA), pp 41-53.
21. R. C. Wills, G. R. V. Hammond, PI(4,5)P2: signaling the plasma membrane. *Biochem J* **479**, 2311-2325 (2022).
22. J. C. Phillips *et al.*, Scalable molecular dynamics on CPU and GPU architectures with NAMD. *J Chem Phys* **153**, 044130 (2020).
23. I. Y. B.-S. D.A. Case, S.R. Brozell, D.S. Cerutti, T.E. Cheatham, III, V.W.D. Cruzeiro, T.A. Darden,, D. G. R.E. Duke, M.K. Gilson, H. Gohlke, A.W. Goetz, D. Greene, R Harris, N. Homeyer, Y. Huang,, A. K. S. Izadi, T. Kurtzman, T.S. Lee, S. LeGrand, P. Li, C. Lin, J. Liu, T. Luchko, R. Luo, D.J., K. M. M. Mermelstein, Y. Miao, G. Monard, C. Nguyen, H. Nguyen, I. Omelyan, A. Onufriev, F. Pan, R., D. R. R. Qi, A. Roitberg, C. Sagui, S. Schott-Verdugo, J. Shen, C.L. Simmerling, J. Smith, R. SalomonFerrer, J. Swails, R.C. Walker, J. Wang, H. Wei, R.M. Wolf, X. Wu, L. Xiao, D.M. York and P.A. Kollman (2018) AMBER 2018. University of California, San Francisco.
24. P. T. Nguyen, K. R. DeMarco, I. Vorobyov, C. E. Clancy, V. Yarov-Yarovoy, Structural basis for antiarrhythmic drug interactions with the human cardiac sodium channel. *Proc Natl Acad Sci U S A* **116**, 2945-2954 (2019).
25. K. R. DeMarco *et al.*, Molecular determinants of pro-arrhythmia proclivity of d- and l-sotalol via a multi-scale modeling pipeline. *J Mol Cell Cardiol* **158**, 163-177 (2021).
26. J. Huang *et al.*, CHARMM36m: an improved force field for folded and intrinsically disordered proteins. *Nat Methods* **14**, 71-73 (2017).
27. J. B. Klauda *et al.*, Update of the CHARMM all-atom additive force field for lipids: validation on six lipid types. *J Phys Chem B* **114**, 7830-7843 (2010).
28. Z. Li, R. M. Venable, L. A. Rogers, D. Murray, R. W. Pastor, Molecular dynamics simulations of PIP2 and PIP3 in lipid bilayers: determination of ring orientation, and the effects of surface roughness on a Poisson-Boltzmann description. *Biophys J* **97**, 155-163 (2009).
29. W. L. C. Jorgensen, J.; Madura, J. D.; Impey, R. W.; Klein, M. L., Comparison of simple potential functions for simulating liquid water. *The Journal of Chemical Physics* **79**, 926-935 (1983).
30. O. S. Smart, J. G. Neduvelil, X. Wang, B. A. Wallace, M. S. Sansom, HOLE: a program for the analysis of the pore dimensions of ion channel structural models. *J Mol Graph* **14**, 354-360, 376 (1996).
31. N. Michaud-Agrawal, E. J. Denning, T. B. Woolf, and O. Beckstein. MDAnalysis: A Toolkit for the Analysis of Molecular Dynamics Simulations. J. Comput. Chem. 32 (2011), 2319–2327. doi:10.1002/jcc.21787
32. W. Humphrey, A. Dalke, K. Schulten, VMD: visual molecular dynamics. *J Mol Graph* **14**, 33-38, 27-38 (1996).

33. E. F. Pettersen *et al.*, UCSF Chimera--a visualization system for exploratory research and analysis. *J Comput Chem* **25**, 1605-1612 (2004).
34. E. F. Pettersen *et al.*, UCSF ChimeraX: Structure visualization for researchers, educators, and developers. *Protein Sci* **30**, 70-82 (2021).
35. T. Tubiana, J. C. Carvaillo, Y. Boulard, S. Bressanelli, TTClust: A Versatile Molecular Simulation Trajectory Clustering Program with Graphical Summaries. *J Chem Inf Model* **58**, 2178-2182 (2018).
